# Supplementary figures and images for: Barriers in care for children with life-threatening conditions: a qualitative interview study in the Netherlands
Source: BMJ Open. 2020 Jun 28;10(6):e035863. doi: 10.1136/bmjopen-2019-035863 (PMC7322336; doi:10.1136/bmjopen-2019-035863)

Appendix: Topic guide

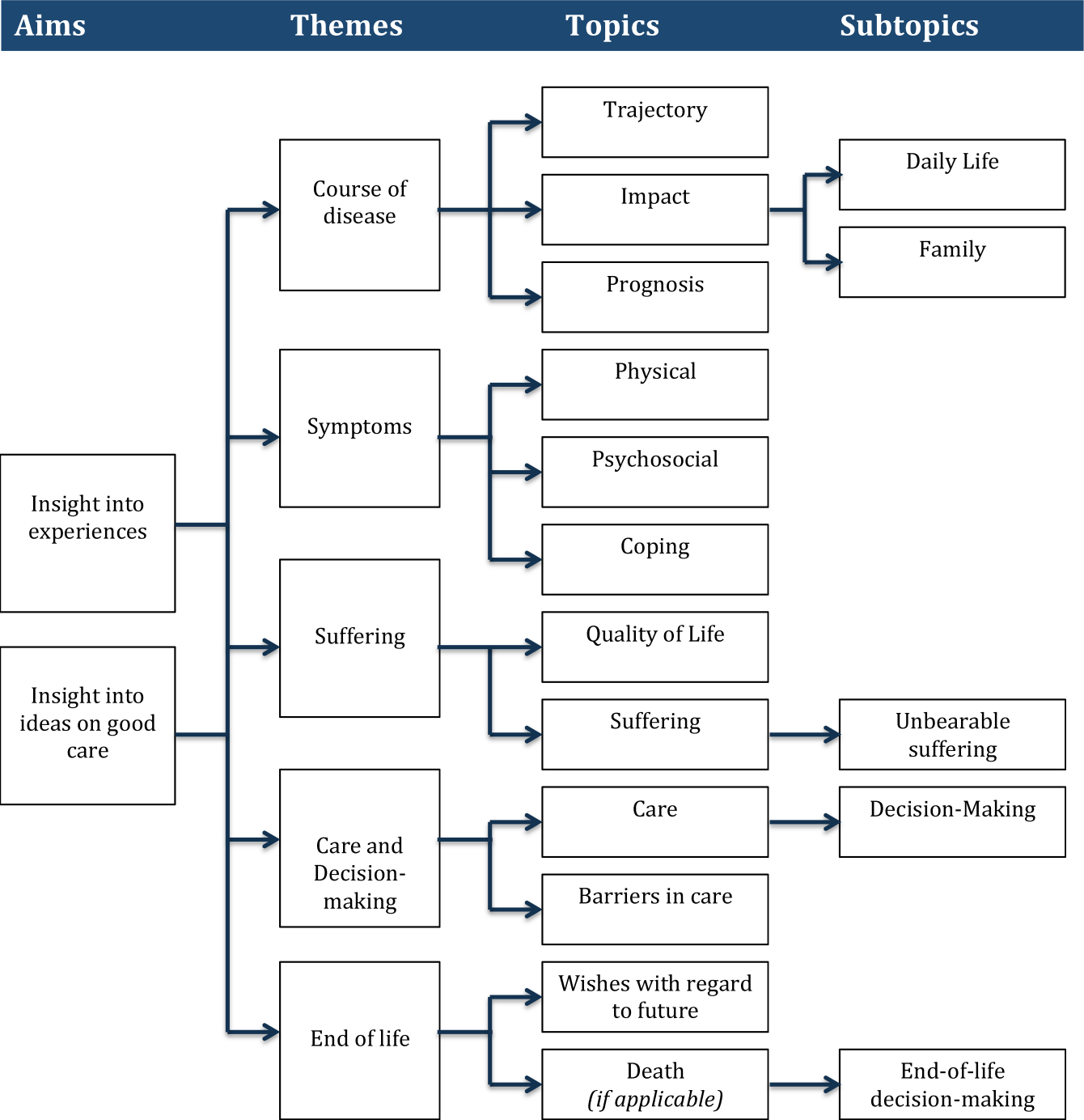

Supplement: Supplementary data [file bmjopen-2019-035863supp001.pdf]
