# Supplementary material for: Barriers in care for children with life-threatening conditions: a qualitative interview study in the Netherlands
Source: BMJ Open. 2020 Jun 28;10(6):e035863. doi: 10.1136/bmjopen-2019-035863 (PMC7322336; doi:10.1136/bmjopen-2019-035863)
Supplement: Author's manuscript [file bmjopen-2019-035863.draft_revisions.pdf]

BMJ Open is committed to open peer review. As part of this commitment we make the peer review history of every article we publish publicly available.

When an article is published we post the peer reviewers' comments and the authors' responses online. We also post the versions of the paper that were used during peer review. These are the versions that the peer review comments apply to.

The versions of the paper that follow are the versions that were submitted during the peer review process. They are not the versions of record or the final published versions. They should not be cited or distributed as the published version of this manuscript.

BMJ Open is an open access journal and the full, final, typeset and author-corrected version of record of the manuscript is available on our site with no access controls, subscription charges or pay-per-view fees (http://bmjopen.bmj.com).

If you have any questions on BMJ Open's open peer review process please email info.bmjopen@bmj.com

BMJ Open

They are Persons, not Symptoms: Parents Express Barriers in Care for Children With Life-Threatening Conditions

Journal:	*BMJ Open*
Manuscript ID	bmjopen-2019-035863
Article Type:	Original research
Date Submitted by the Author:	19-Nov-2019

Complete List of Authors:	Brouwer, Marije; Universitair Medisch Centrum Groningen, pediatrics<br>Maeckelberghe, Els; Universitair Medisch Centrum Groningen, Institute for Medical Education FC 40<br>van der Heide, Agnes; Erasmus MC, University Medical Center Rotterdam, Clinical Microbiology<br>Hein, Irma; Academic Medical Center: Amsterdam, psychiatry<br>Verhagen, Eduard; University Medical Center Groningen, University of Groningen, Dept of Pediatrics

Keywords:	PALLIATIVE CARE, MEDICAL ETHICS, Paediatric palliative care < PALLIATIVE CARE

SCHOLARONE™
Manuscripts

I, the Submitting Author has the right to grant and does grant on behalf of all authors of the Work (as defined in the below author licence), an exclusive licence and/or a non-exclusive licence for contributions from authors who are: i) UK Crown employees; ii) where BMJ has agreed a CC-BY licence shall apply, and/or iii) in accordance with the terms applicable for US Federal Government officers or employees acting as part of their official duties; on a worldwide, perpetual, irrevocable, royalty-free basis to BMJ Publishing Group Ltd ("BMJ") its licensees and where the relevant Journal is co-owned by BMJ to the co-owners of the Journal, to publish the Work in this journal and any other BMJ products and to exploit all rights, as set out in our licence.

The Submitting Author accepts and understands that any supply made under these terms is made by BMJ to the Submitting Author unless you are acting as an employee on behalf of your employer or a postgraduate student of an affiliated institution which is paying any applicable article publishing charge ("APC") for Open Access articles. Where the Submitting Author wishes to make the Work available on an Open Access basis (and intends to pay the relevant APC), the terms of reuse of such Open Access shall be governed by a Creative Commons licence – details of these licences and which Creative Commons licence will apply to this Work are set out in our licence referred to above.

Other than as permitted in any relevant BMJ Author's Self Archiving Policies, I confirm this Work has not been accepted for publication elsewhere, is not being considered for publication elsewhere and does not duplicate material already published. I confirm all authors consent to publication of this Work and authorise the granting of this licence.

For peer review only

They are Persons, not Symptoms: Parents Express Barriers in Care for Children With Life-Threatening Conditions

MARIJE BROUWER, ELS MAECKELBERGHE, AGNES VAN DER HEIDE, IRMA HEIN, and EDUARD VERHAGEN

Marije A. Brouwer, MA
m.a.brouwer@umcg.nl
Department of paediatrics, University of Groningen, University Medical Center Groningen, Groningen, the Netherlands

dr. Els L.M. Maeckelberghe
e.l.m.maeckelberghe@umcg.nl
Institute for Medical Education, University of Groningen, University Medical Center Groningen, Groningen, the Netherlands

prof. Agnes van der Heide
a.vanderheide@erasmucmc.nl
Department of Public Health, Erasmus medical Center, Rotterdam, the Netherlands

dr. Irma M. Hein
i.hein@debascule.nl
Department of psychiatry, Academic Medical Center, Amsterdam, the Netherlands

prof. A.A.E. Eduard Verhagen
a.a.e.verhagen@umcg.nl
Department of paediatrics, University of Groningen, University Medical Center Groningen, Groningen, the Netherlands

Address correspondence to:
Marije Aafke Brouwer
University Medical Center Groningen, Department of Pediatrics,
tnv A.J.Heinstra/M.A.Brouwer.
Antwoordnummer 333, 9700 VB Groningen
mail: m.a.brouwer@umcg.nl
phone: 0031-621102049

Word count (excluding abstract, references, and quotes): 3244
Word count (excluding abstract and references, including quotes): 4033

For peer review only

Abstract.

Objective: To identify barriers, as perceived by parents, in good care for children with life-threatening conditions.

Design: In a nationwide qualitative study, we held in-depth interviews with parents of children (aged one to 12 years) who were living with a life-threatening illness or who had died after a medical trajectory (a maximum of five years after the death of the child). Sampling was aimed at obtaining maximum variety for a number of factors. The interviews were transcribed and analysed.

Setting: The Netherlands.

Participants: 64 parents of 44 children.

Results: Parents identified six categories of difficulties that create barriers in care for children with a life-threatening condition. The first barrier involves a lack of empathy in communication and an absence of conversations about the uncertain future of the child. The second barrier is lack of continuity in care, where bureaucratic obstacles stand in the way. Third are obstacles with regard to the participation of parents in medical decision-making. The fourth perceived barrier concerns end-of-life decision-making, where parents wish for more options to make end-of-life decisions, including euthanasia. The fifth is a lack of attention of health care professionals for the family's welfare, and the sixth barrier that parents described is the medical setting failing to recognize the child as a person, but seeing them as carriers of symptoms.

Conclusions: The barriers as perceived by parents focused almost without exception on non-medical aspects: patient-doctor relationships, communication, decision-making, including end-of-life decision-making, and organization. The perceived barriers indicate that care for children with a life-threatening condition focuses too much on symptoms and not enough on the human beings behind those symptoms.

Article summary

Limitations:

-The study focuses on young children (aged one to 12 years old) and the results cannot be generalized to adolescents or neonates.

- We did not include the perspectives of physicians.

For peer review only

Strengths:

- Size of the study: 64 participants and over 110 hours of qualitative data on audio.
- The variety: contrary to existing research, this study includes a wide variety of medical conditions in various stages of their illness, providing a much needed broad perspective on care given to children living with life-threatening conditions.

For peer review only

What is already known about this topic:

Healthcare providers describe as the main barriers is palliative care: communication, an uncertain prognosis, time constraints and lack of education

Parents describe communication, lack of care for the family and lack of bereavement care as barriers, but these studies are limited to the terminal phase and/or specific life-threatening illnesses.

What this study adds:

-This study shows that barriers experienced by parents in care for children with life-threatening conditions and uncertain futures are related to 1) communication, 2) a lack of continuity in care, 3) a lack of shared decision-making, 4) end-of-life decision-making 5) a lack of attention for the family's welfare, 6) failing to see the child as a person.

- The parents' wish to see the child as a person sheds new light on the relationship between medical professionalism and detachedness.

For peer review only

Introduction.

Parents and physicians caring for children with life-threatening conditions face multiple challenges in providing the best care for children. Care for children whose futures and chances of survival are uncertain is often a complex trajectory, where curative and palliative care entwines.[1, 2] Curative care goals – to cure/prolong life – and palliative care goals – to relieve suffering – sometimes collide, complicating care. Care for these children is further complicated because decisions about the proportionality of life-prolonging treatments, and sometimes end-of-life decisions, are considered part of palliative care [3, 4]

Life-threatening conditions are defined as conditions for which curative treatment may be feasible, but for which this treatment could also fail, leading to a possibility of dying.[5] Care for children with life-threatening conditions can include aspects of both curative and palliative care at the same time.[2] Healthcare providers are not always confident about their skills in providing palliative care.[6] Communication, an uncertain prognosis, time constraints and a lack of education are the main barriers in paediatric palliative care experienced by healthcare providers.[7-11] Studies on barriers experienced by parents show that communication is a major barrier for them.[12-17] Other frequently mentioned barriers are a lack of care for the family[12-13] and bereavement care.[17]

But knowledge of parents' experiences of barriers to good care is limited in at least two ways. First, it focuses mainly on specific health conditions, particularly on oncology,[14,15] and second, it focuses mainly on children receiving palliative care, often with a certain, lethal prognosis.[12-13,16-17] However, not all children who have life-threatening conditions receive palliative care, or are diagnosed with a lethal prognosis.[18-20]

To provide insight into barriers to care and decision-making for children living with life-threatening conditions, we conducted a qualitative study throughout the Netherlands. We interviewed parents who had experience in caring for a child with a life-threatening condition to identify what they regarded as barriers to both good care and good decision-making.

Methods.

In a large-scale, nationwide qualitative study we interviewed parents of children, either living with a life-threatening or terminal condition, or who had died after a medical trajectory (a maximum of five years before the date of the interview). Children were aged between one and 12 years old and had a variety of life-threatening conditions.

Recruitment.

Participants were recruited between November 2016 and October 2018. To recruit participants, all Dutch paediatricians received an invitation from the Paediatric Association of the Netherlands (NVK) to invite potential parents to join the research.[21] A website with information about the study was created to inform potential participants.[22] Parent support groups in oncology, cardiology, metabolic and neuromuscular diseases and palliative care helped to reach potential participants through their online platforms. To ensure maximum variety in ethnic background we recruited through physicians with expertise in treating patients with different cultural backgrounds. Sampling was aimed at obtaining maximum variety in terms of health condition, age, cultural background, parental level of education and place of residence. Three participants dropped out due to scheduling difficulties.

Interviews.

Parents were interviewed in face-to-face in-depth interviews, usually held at the parent's place of residence. The first author (MA, PhD student, female) conducted the interviews after following formal training. Parents did not know the interviewer before the study. All participants were given the choice to conduct the interview alone or together with a co-parent. The interviews were recorded on audio and subsequently transcribed verbatim. The average duration of the interviews was two hours. Participants were given the opportunity to revise their manuscripts.

Analysis.

For this article, we selected quotes concerning perceived barriers in care. For reasons of practicality in handling the approximately 2,500 pages of transcripts, the first author gathered all codes on communication in parent interviews. All authors read the selection to become familiarized with the content. The first author further focused on parents' perceptions of barriers in care in the selection of interview fragments, and this was reviewed by all authors.

For peer review only

A primary thematic analysis was performed using the constant comparative approach.[23] Coding was performed by the first two authors and reviewed by all authors. Differences were settled by discussion until consensus was achieved.

Patient and public involvement

Family of patients were involved in the study by means of an advisory board. During the study, biannual meetings were held with an advisory board of parents, physicians and researchers, who provided feedback on the design of the study, the analysis of the interviews, and the findings of the study.

For peer review

Results.

Participants. We interviewed 64 parents of 44 children. Our participants came from all over the Netherlands. All children received, or had received, care in at least one university medical centre, often combined with care in one or more local hospitals. All eight university hospitals in the Netherlands, as well as over 20 local hospitals, were represented in the study. Characteristics of children and parents are shown in Tables 1,2 and in Figure 3.

Table 1. Characteristics of children (n=44)

Status, deceased	27 (61.4%)
Gender, male	20 (45.5%)
Age (years)	
1-3	15 (34.0%)
4-6	8 (18.2%)
7-9	9 (20.5%)
10-12	12 (27.3%)
Diagnosis	
Malignancies	18 (40.9%)
Neurological/metabolic	17 (38.6%)
Cardiovascular	4 (9.1%)
CNS	3 (6.8%)
Other	1 (2.2%)
Undiagnosed	1 (2.2%)

Table 2. Characteristics of parents (n=64 interviewed parents, 42 couples)

Relationship status	
Married/Together	34 (81.0%)
Level of education, mothers (N=42)	
Low-level education	1 (2.4%)
Mid-level education	15 (35.7%)
Higher education	14 (33.3%)
University	12 (28.6%)
Level of education, fathers (N=42)	
Low-level education	4 (9.5%)
Mid-level education	15 (35.7%)
Higher education	14 (33.3%)
University	9 (21.4%)
Ethnicity of parents, according to the participants (N=42)	
Dutch	36 (85.7%)
Mixed	6 (14.3%)
Religious/ spiritual beliefs (N=64)	
None	39 (60.9%)
Christian	19 (29.7%)
Other	6 (9.4%)
Family composition	
1 child	8 (19.0%)
2 children	22 (52.4%)
3 children	10 (23.8%)
4 or more children	2 (4.8%)

For peer review only - http://bmjopen.bmj.com/site/about/guidelines.xhtml

Figure 3. Participants' places of residence

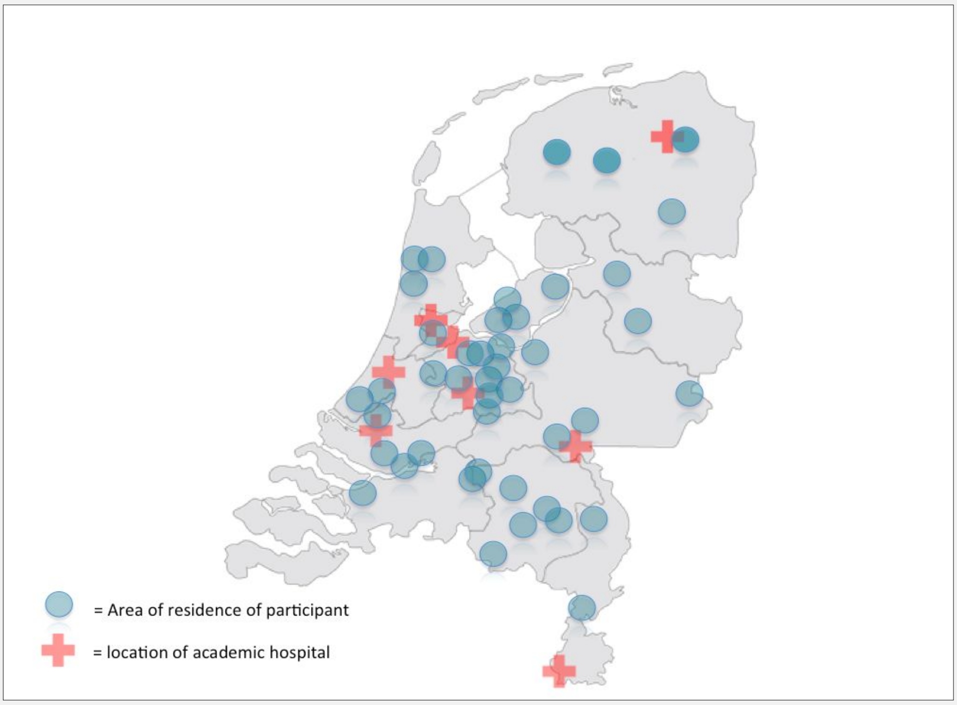

Experienced barriers. We identified six major barriers in care for children with life-threatening conditions: 1) communication, 2) a lack of continuity in care, 3) a lack of shared decision-making, 4) end-of-life decision-making, 5) a lack of attention for the family's welfare, 6) failing to see the child as a person.

Barrier 1: Communication.

Parents univocally felt that the wish to receive complete and open information, and the absence thereof, was a significant barrier to good care. Most experiences regarding communication involved either a perceived lack of openness about the prognosis or a lack of empathy in communication.

1a. Openness about the prognosis. Telling a parent that their child may not survive might seem like a message that parents would not like to receive, but many parents (both bereaved parents and non-bereaved parents) stressed that they had wished for open conversations about their child's future. Not talking about the prognosis creates uncertainty and makes the topic a taboo. They underlined the importance to openly talk about this, especially when the prognosis is uncertain.

(F02): 'In the first few years, you still have the hope that things might change, but after a few years, you realize that almost all of that hope has gone. It would be great if physicians would take you by the hand and say: "We have to accept that this is what it is, and that things are probably not going to get better." And ask you as a parent: "What are your thoughts on that? Would you, despite that prognosis, like to keep on trying new treatments? Or do you think it is better not to do that?"'

Parents specifically needed openness about the prognosis to discuss the proportionality of treatment and possible end-of-life decisions. In a few cases, the topic of dying was only mentioned a day before the death of their child.

1b. Insensitive communication. Parents experienced a lack of empathic communication. They felt that the friction between healthcare professionals doing their daily jobs and families going through a once-in-a-lifetime event was characteristic for the lack of sympathy that they experienced.

(M27): '[the lady who came to explain the procedure of palliative sedation] was so insensitive. She just explained all the technicalities, with a broad smile. And I remember thinking: "It's not like I am buying bread from you!" (…) It was as if she thought: "let's get this over with", as if there was not an entire life, an entire family, an entire world affected by the procedure that she was explaining.'

But experienced barriers in communication were not limited to interactions with healthcare providers or hospital settings.

(F16): 'Before we had even held the funeral, we received a letter from the local authority asking us to return our special parking permits as soon as possible. Just like that, without any condolences. Bizarre.'

Barrier 2: Organization.

With regard to organization, two themes emerged: continuity of care and bureaucracy.

2a. Continuity of care. Parents described having to deal with many different care providers, either individual professionals or organizations, which made it difficult to guarantee

For peer review only

continuity of care. Agreements made with one provider did not always dovetail well with the care that they received from other care providers.

> (F03): 'There was a physician on duty, and when we updated him on the 'do not resuscitate' agreements that we had made with our regular physician, he said: "No, I'm not going to do that. It is my shift, and my responsibility, and I'm not going to do that." You take all this time to make decisions with your doctor, and then another doctor, who happens to be on duty and doesn't know your child or the file, who has never had a conversation with you, simply states: "I'm responsible, so I determine what happens."'

2b. Bureaucracy. Parents particularly experienced bureaucratic barriers to good care in situations where they had to deal with a multitude of healthcare-related organizations.

> (M31) 'When the neurologist prescribes some sort of medicine, he has to send it by fax to the pharmacist, he can't send it by email. And if there is even one comma in the wrong place, the pharmacist won't prescribe the medicine that my son needs.'

Several parents expressed the need for a case manager, who could bridge the gaps between parents, different healthcare providers and subsidy providers. Notably, this barrier was also mentioned by parents who had received palliative care from teams that already worked with case managers. While more and more paediatric palliative teams in the Netherlands are appointing case managers to families dealing with a life-threatening illness, the question arises as to how they can be used in the most effective manner.

Barrier 3: Decision-making.

The third barrier concerned participation in decision-making. There was a lot of variation regarding the involvement of parents in decision-making. Some parents appreciated being able to participate in the decision-making process.

> (M15): '[when we had to decide whether to go for radiotherapy or not] our doctor said: "Whatever your decision is, there is no wrong answer, and I will stand by your choice."'

Other parents described that they were not as involved in decision-making as they would have liked, both for decisions made during curative treatment and for those made in the terminal

For peer review only

phase. One parent described that the decision to withdraw life-sustaining treatment for their child was made by a neurologist who had never spoken to them.

> (M27): 'The neurologist never spoke to us about his motivations to [withdraw treatment] and start the death of our son. He came in, made the observation right in front of us, but only spoke in private to our paediatrician, and left.'

Barrier 4. End-of-life decision-making.

For many participants, discussions about the proportionality of treatment and end-of-life decision-making were major themes in the care for their child. A significant number of parents had doubts about the appropriateness of continued treatment or felt that it was wrong that parents themselves had to initiate a conversation about end-of-life decision-making.

Additionally, several parents expressed a wish for more legal possibilities for active life-ending in order to relieve their child's suffering and grant their child a dignified end of life. In the Netherlands, active life-ending for children – sometimes referred to as paediatric euthanasia – is not legal for children aged between one and 12 years old.[24] Withholding or withdrawing treatment and palliative sedation are considered permitted medical practices.[25]

For the majority of the children in the study, end-of-life decisions were made, ranging from withholding or withdrawing life-prolonging or life-sustaining treatment to palliative sedation. Nevertheless, in several cases, parents still felt the need for an option to make end-of-life decisions. In most cases, their need came from a situation in which the condition of their child was physiologically stable and the physicians could not justify the withdrawal of treatment or palliative sedation.

> (M02) 'Physicians told us that if they had to [give her] so much morphine to ease her pain that the morphine itself would be the cause of her death, it would be considered active life-ending, which is not allowed for children who cannot ask for it themselves. But we would want that for her, because we have the feeling that the only thing that can bring her comfort and peace (emotional) will be death.'

In other situations, parents felt a need for active life-ending because the existing options were unable to grant their child a dignified end-of-life.

For peer review only

> (M07): 'If we all know that the palliative sedation is going to result in his death, why does he have to go through these final days of suffering? Why does it have to be so... undignified?'

Barrier 5: Family care.

A life-threatening illness does not only affect children, but also their families. Parents felt that they and their families were not always supported during and after the illness of their child. Siblings in particular were often overlooked.

> (M20): 'As a parent you get a little support, but for brothers and sisters there is very little to absolutely no help at all. They have to figure it out for themselves, it seems as though they are forgotten.'

While support for the family is one of the aims of paediatric palliative care,[25] and all Dutch hospitals have psychologists, social workers and/or palliative care teams to provide this, it seems that this support is not always offered to parents or did not function as well as parents had hoped. However, for most parents, the need was not so much about offering psychological support as it was about empathy in their contact with the healthcare providers that they came across.

> (M05) 'What they don't always realize is that the hospital also becomes your social world; the contact with doctors, the chats with nurses.'

Barrier 6: The child behind the symptoms.

The final barrier expressed by parents was a wish for healthcare providers to understand the fact that patients are persons, not merely carriers of certain symptoms. They explained that healthcare professionals often failed to evaluate the impact of the condition or the medical interventions on the child's daily life. This barrier had three different aspects: 1) a lack of attention for how a child might understand and cope with treatment and illness, 2) failure to see the complexity of the child as a whole and, finally, 3) overemphasizing the child as a patient instead of as a whole person.

For peer review only

6a. Children's understanding of illness. One specific characteristic of paediatric care is the attention given to how children understand and cope with their condition and the associated treatment. Parents underlined that this is important both for 'bigger questions' and for simple medical interventions.

> (M04) 'Hospitals are busy with production. So, a nurse comes in, and basically jumps on the child to take his blood pressure, or give an injection, without paying attention to what it does to him (…) And I often wonder: How hard can it be to ask yourself beforehand how to approach this individual child, so that he understands the interventions?'

6b The *child* and the illness. Many of the children with life-threatening conditions receive treatment from various specialists. Parents described how specialists sometimes failed to look beyond the specific symptoms that they were treating and see the child and the complexity of their condition as a whole.

> (M29): 'We had an incident because my son had fallen, and the dental surgeon took one look at his teeth and said: "Those teeth have got to go." And I said: "But what then? He already has problems swallowing." And he said casually: "He'll need a feeding tube." Not caring what it would mean to him, and our lives, to make that decision. And I don't think he even saw the boy who was sitting there, who I love so much, and who I am so worried about.'

6c. The patient instead of the person. The final barrier identified was the overemphasis of the child as a patient, at the cost of the child as a person. Parents described how medical treatment sometimes overemphasized the child as a patient and that there was little attention for the child as a person, impeding the child's quality of life. Parents stressed that especially for ill children keeping up a normal life became very important: going to school, not standing out, feeling like an ordinary child. Daily activities that did not focus on the child's illness, but on the child as an ordinary person, became cherished. As this mother remembers:

> (M14): 'It was the highlight of his day, when his teacher would come; solving math problems (laughs).'

This barrier also influenced the readiness of a child to communicate about their illness. Many children did not want to talk about the illness, a few even forbidding their parents to talk about it with others. Seeing the child too much as a patient, either by focussing merely on the

For peer review only

child's illness, or by trying to make them feel special, might deprive children of living a normal life.

Both barriers five and six refer to what parents described as their wish for a more human-centred approach to care, which also implied an empathic connection between professionals and parents/patients. According to the parents, some physicians perceived this attitude as unprofessional:

> (M20): 'One physician had a big impact on me. We had to go home, and she said goodbye to my son. She started crying and apologized: "I'm so sorry, I'm being very unprofessional."'

For parents, however, this kind of connection was much appreciated and in fact seen as a sign of professionalism.

> (M20, continued): 'And I said: "No, right now, you are human." I wonder if she knows that of all those doctors, she is the only one that crosses my mind frequently. Because she cared.'

Discussion.

Main findings. This qualitative study gives insight into parents' perceived barriers in care for children with life-threatening conditions. The barriers experienced by parents focused almost without exception on the non-technical skills in medicine: attention for children, empathy, communication, decision-making and organization. The absence of perceived barriers on the execution of symptomatic care is notable. We summarized the barriers in Table 4.

For peer review only

Table 4. Barriers in paediatric palliative care, as experienced by parents.

**Barrier 1. Communication**
1a. Communication lacks empathy.
1b. Absence of conversations about the future and possible prognosis.
**Barrier 2: Organization**
2a. Barriers in continuity of care.
2b. Healthcare structures create bureaucratic obstacles.
**Barrier 3. Participation in decision-making**
**Barrier 4. End-of-life decision-making**
**Barrier 5. The family behind the symptoms is not seen**
5a. Impact on parents and siblings.
5b. Lack of support after the death of the child.
**Barrier 6. The child behind the symptoms is not seen**
6a. Lack of attention for how a child might understand treatment and illness.
6b. Failure to see the complexity of the child as a whole.
6c. Overemphasizing the patient instead of the person.

With respect to the first barrier, communication, other studies report communication as a barrier for both physicians [7-8,11,26-30] and parents.[12-16,31-33] Our results underline the importance of openness as a requirement for decision-making, and adds that communication is not limited to hospital settings. A possible future development for paediatric palliative care is to extend communication training to all professionals dealing with children with life-threatening conditions, not merely hospital workers.

To reduce organizational barriers, parents suggested introducing case managers that might help parents and children with bridging the gaps between different healthcare professionals and in dealing with bureaucratic difficulties. Over the past year, several paediatric palliative care teams in the Netherlands have introduced such case managers, with the aim of trying to reduce this barrier. However, it is worth mentioning that in current practice, palliative care is predominantly limited to terminally ill children, and consequently, these palliative care services also remain limited to the terminal phase. However, issues with the continuity of care and with bureaucracy may arise long before this phase.

With regard to the third barrier, decision-making, the involvement of parents in decision-making varied. Previous studies also show that parents are not always involved in decision-

For peer review only - http://bmjopen.bmj.com/site/about/guidelines.xhtml

making.[34-35] Shared decision-making has been an important movement in both paediatrics and medicine in general over the last decade.[36-37] Dutch regulations underline the importance of making parents, and – if possible – even children themselves, part of the decision-making process.[38] The fact that parents often feel excluded may be an incentive to further investigate why parents are not always included, and what is needed to achieve this.

The perceived need for end-of-life decisions is a barrier that, to our knowledge, has not previously been mentioned in studies researching barriers in care for children with life-threatening conditions. In contrast, other studies mention parents' discomfort with making end-of-life decisions such as withholding or withdrawing treatment.[39] Although the Netherlands is known for having a legal regulation of euthanasia and end-of-life decision-making in adults and newborns,[24,40-41] the current regulations for children aged between one and 12 years old are similar to those in most other countries. Withholding and withdrawing treatment and palliative sedation are permitted, but active life-ending (euthanasia) is not. Following the Belgian legalization of euthanasia for competent minors, there has been a debate in the Netherlands about whether regulations on euthanasia should be broadened to allow active life-ending for children younger than 12 in cases of unbearable and hopeless suffering.[43-44]

Parents perceived a lack of care for the family. Family care is one of the goals of palliative care[8] and is arguably a facility that all parents and siblings of the child should receive.[18] Previous studies highlight the importance of family support, including bereavement care.[12-13,17]

Previous studies have underlined that care for young children includes attention for a child's development.[18,44] Our study adds that this does not only mean seeing the child as a whole, with both physical, social and emotional aspects, but that it also means seeing the child as a *person*. From the perspective of parents, care for children with life-threatening conditions focuses too much on symptoms and not enough on the human beings behind the symptoms. This raises the question of whether such an attitude should be part of medical professionalism.

Medical professionalism: dehumanization?

For peer review only - http://bmjopen.bmj.com/site/about/guidelines.xhtml

Medicine and emotional intimacy seem to have a difficult relationship. Can a professional working in paediatric palliative medicine be expected to build an empathic, intimate relationship with all of their patients? Some authors argue that what makes a good doctor is, in essence, expertise and knowledge, not empathy; that good doctor may be stand-offish or surly, as long as they treat a symptom adequately.[45] Intimacy is a tool needed to detect the needs of a patient, but should be used very carefully, otherwise a doctor might 'lose himself''.[45-47] Nevertheless, empathy benefits patients.[48] The view of detachedness as a professional quality is often – although not always explicitly – reiterated throughout medicine. There is a tension between this viewpoint of professional detachedness and that of empathic intimacy, or human-to-human contact, that parents ask for.

What parents seem to miss in current practise is the other connotation of the word 'care': to care about. We cannot expect physicians or nurses to become emotionally attached to every patient that they meet, but this is not what parents seem to miss in current care. What they miss in care is not the lack of showing emotion, but the affirmation that all parties – children, family and physicians – are in fact human and that the illness affects lives, not monitors. This attitude can be implemented into standard care for children with life-threatening conditions.

By its goal –improving quality of life by relieving suffering–[2] paediatric palliative care shifts its focus from the illness and symptom towards the patient. The experienced barriers indicate that this shift is possibly insufficiently translated into care. Perhaps it is time to consider how we should evaluate professional detachedness in the light of the goals of palliative care. Above all, policies and guidelines should not merely guide professionals on the technical aspects of symptom treatment, but aid them in topics of professional intimacy as well.

Strengths and limitations.

Our study has some limitations. First, our study focused on young children (aged one to 12 years old) and the results cannot be generalized to adolescents or neonates. Second, although we put considerable effort into recruiting participants with different cultural and religious backgrounds, the success in achieving this variation was limited. Finally, we did not include the perspectives of physicians, in order to solely present the barriers perceived by parents. Although the parents' own experiences are the experiences that they live by, and that represent their reality, they do not present a complete overview alone. In order to fully evaluate care for children with life-threatening conditions, the reality of the physicians, nurses and other involved parties should also be described.

For peer review only

The strengths of this study are the size of the study. We held in-depth interviews with 64 participating parents, and over 110 hours of qualitative data on audio were collected. This makes the study unique in its size. A second strength is the variety. Most existing research is limited either by medical diagnosis or hospital where care is administered. In this study we interviewed parents from children with various illnesses, in various stages of their illness, receiving care from various hospitals, providing a much needed broad perspective on care given to children living with life-threatening conditions.

Acknowledgements.

The authors would like to thank the participants for sharing their experiences. We also thank Veerle van de Wetering, and the members of the advisory group for their help in shaping the study, and their input during the analysis of the study. We thank Suzanna Miedema-Wilsterman for her efforts in recruiting participants for this study.

Contributorship statement

The authors of this article, Marije Brouwer, Els Maeckelberghe, Agnes van der Heide, Irma Hein and Eduard Verhagen, designed and conducted this study, including study design, data collection and data analysis. Marije Brouwer conducted the interviews. The analysis was performed by Marije Brouwer and Els Maeckelberghe, and checked by all authors. All authors contributed to the final manuscript.

Ethical approval

The study was reviewed and deemed exempt by the medical ethical committee of the University Medical Center Groningen. Number: METC2016. 223.

Competing Interest statement.

This work was supported by the Dutch Ministry of Health, Welfare and Sport. Grant number 324717 (560019968)]. The authors have no further conflict of interest or financial relations to disclose.

Dissemination Statement.

All participants that contributed to this study are frequently updated on the reporting of the study. We plan to disseminate the results to the participants of the study.

For peer review only

Data Sharing Statement

No additional Data Available.

Exclusive licence statement.

The Corresponding Author hereby declare to have the right to grant on behalf of all authors and does grant on behalf of all authors, a worldwide licence to the Publishers and its licensees in perpetuity, in all forms, formats and media (whether known now or created in the future), to i) publish, reproduce, distribute, display and store the Contribution, ii) translate the Contribution into other languages, create adaptations, reprints, include within collections and create summaries, extracts and/or, abstracts of the Contribution and convert or allow conversion into any format including without limitation audio, iii) create any other derivative work(s) based in whole or part on the on the Contribution, iv) to exploit all subsidiary rights to exploit all subsidiary rights that currently exist or as may exist in the future in the Contribution, v) the inclusion of electronic links from the Contribution to third party material where-ever it may be located; and, vi) licence any third party to do any or all of the above. All research articles will be made available on an open access basis (with authors being asked to pay an open access fee). The terms of such open access shall be governed by a Creative Commons licence—details as to which Creative Commons licence will apply to the research article are set out in our worldwide licence referred to above.

References

1. Mack JW, Wolfe J. Early integration of pediatric palliative care: for some children, palliative care starts at diagnosis. Curr Opin Pediatr 2006;18:10-4. doi:10.1097/01.mop.0000193266.86129.47 pmid:16470155
2. World Health Organization. WHO definition of palliative care. [cited 2019 Oct 08]. Available from: https://www.who.int/cancer/palliative/definition/en/
3. Brouwer M, Maeckelberghe E, De Weerd W, et al. Quality of Living and Dying: Pediatric Palliative Care and End-of-Life Decisions in the Netherlands. Camb Q Healthc Ethics 2018;27:376-84. doi:10.1017/S0963180117000767 pmid:29845907
4. Paediatric Association of the Netherlands (NVK). Richtlijn Palliatieve Zorg voor Kinderen. 2018 [cited 2019 Oct 08]. Available from: http://richtlijnendatabase.nl/richtlijn/palliatieve_zorg_voor_kinderen
5. International Children's Palliative Care Network (ICPCN) What is children's palliative care? 2010. [cited 2019 Oct 08]. Available from: http://www.icpcn.org.uk/page.asp?section=0001000100080004§ionTitle=What+is+Children%92s+Palliative+Care%3F

6. Sheetz MJ, Bowman MA. Pediatric palliative care: an assessment of physicians' confidence in skills, desire for training, and willingness to refer for end-of-life care. American Journal of Hospice and Palliative Medicine 2008;25:100-5. doi:10.1177/1049909107312592 pmid:18445861
7. Davies B, Sehring SA, Partridge JC, et al. Barriers to palliative care for children: perceptions of pediatric health care providers. J. Pediatr 2008;121:282-8. doi:10.1542/peds.2006-3153 pmid:18245419
8. Beckstrand RL, Rawle NL, Callister L, et al. Pediatric nurses' perceptions of obstacles and supportive behaviors in end-of-life care. American Journal of Critical Care. 2010;19:543-52 doi:10.4037/ajcc2009497 pmid:20026650
9. Gowan D. End-of-life issues of children. Pediatr Transplant 2003;7:40-3. doi:10.1034/j.1399-3046.7.s3.5.x pmid:12603691
10. Bartel DA, Engler AJ, Natale JE, et al. Working with families of suddenly and critically ill children: physician experiences. Arch Pediatr Adolesc Med 2000;154:1127-33. doi:10.1001/archpedi.154.11.1127 pmid:11074855
11. Williams-Reade J, Lamson AL, Knight SM, et al. Paediatric palliative care: A review of needs, obstacles and the future. J Nurs Manag 2015;23:4-14. doi:10.1111/jonm.12095 pmid:23944156
12. Davies B, Connaughty S. Pediatric end-of-life care: Lessons learned from parents. J Nurs Adm 2002;32:5-6. doi:10.1097/00005110-200201000-00001 pmid:11802640
13. Contro N, Larson J, Scofield S, et al. Family perspectives on the quality of pediatric palliative care. Arch Pediatr Adolesc Med 2002;156:14-9. doi:10.1001/archpedi.156.1.14 pmid:11772185
14. Tenniglo LJ, Loeffen EA, Kremer LC, et al. Patients' and parents' views regarding supportive care in childhood cancer. Support Care Cancer 2017;25:3151-60. doi:10.1007/s00520-017-3723-7 pmid:28456909
15. Nyborn JA, Olcese M, Nickerson T, et al. "Don't try to cover the sky with your hands": parents' experiences with prognosis communication about their children with advanced cancer. J Palliat Med 2016;19:626-31. doi:10.1089/jpm.2015.0472 pmid:27115314
16. Hsiao JL, Evan EE, Zeltzer LK. Parent and child perspectives on physician communication in pediatric palliative care. Palliat Support Care 2007;5:355-65. doi:10.1017/S1478951507000557 pmid:18044413
17. Widger K, Picot C. Parents' perceptions of the quality of pediatric and perinatal end-of-life care. Pediatr Nurs 2008;34;53-8.
18. Goldman A. ABC of palliative care: special problems of children. BMJ. 1998;316:49–5. doi: 10.1136/bmj.316.7124.49 pmid:9451269
19. Hynson JL, Gillis J, Collins JJ, et al. The dying child: how is care different? Med J Aust. 2003;179:20–2. doi:10.5694/j.1326-5377.2003.tb05571.x pmid:12964930
20. Mack JW, Wolfe J. Early integration of pediatric palliative care: for some children, palliative care starts at diagnosis. Curr Opin Pediatr 2006;18:10-4. doi:10.1097/01.mop.0000193266.86129.47 pmid:16470155
21. Paediatric Association of the Netherlands (NVK). oproep melden casus levenseindezorg 1-12 jaren. 2016 [cited 2019 Oct 08]. Available from:

https://www.nvk.nl/Nieuws/articleType/ArticleView/articleId/1707/Oproep-melden-casus-levenseindezorg-1-12-jarigen

22. Brouwer M. Maeckelberghe E, Verhagen E. Beslissingen en Zorg voor Kinderen met een Levenbedreigende Aandoening, Project Website. 2017, [cited 2019 Oct 08]. Available from: https://levensbedreigendziekkind.wordpress.com

23. Hennink M, Hutter I, Bailey A. Qualitative research methods. Thousand Oaks, CA, Sage; 2010 Dec 8

24. Government of the Netherlands. Wet toetsing levensbeëindiging op verzoek en hulp bij zelfdoding. [Termination of life on request and assisted suicide (Review Procedures) Act]. 2014. [cited 2019 Oct 08]. Available from: http:// wetten.overheid.nl/BWBR0012410/2014-02-15

25. Paediatric Association of the Netherlands (NVK). Richtlijn Palliatieve Zorg voor Kinderen. 2018. [cited 2019 Oct 08]. Available from: http://richtlijnendatabase.nl/richtlijn/palliatieve_zorg_voor_kinderen

26. Meyer EC, Sellers DE, Browning DM, et al. Difficult conversations: improving communication skills and relational abilities in health care. Pediatr Crit Care Med 2009;10:352-9. doi:10.1097/PCC.0b013e3181a3183a pmid:19325506

27. Hancock K, Clayton J, Parker S, et al. Truth telling in discussing prognosis in advanced life-limiting illnesses: a systematic review. Palliat Med. 2007;21:507-17. doi: 10.1177/0269216307080823 pmid:17846091

28. Crosnier-Schoedel C, Trocmé N, Carbajal R, et al. Pediatrician's experience in announcing bad news. Arch Pediatr. 2018;25:100-6. doi: 10.1016/j.arcped.2017.11.006 pmid:29254736

29. Baile WF, Lenzi R, Kudelka AP, et al. Improving physician—patient communication in cancer care: Outcome of a workshop for oncologists. J Cancer Educ 1997;12:166-73.

30. Coad J, Patel R, Murray S. Disclosing terminal diagnosis to children and their families: Palliative professionals' communication barriers. Death Stud 2014;38:302-7. doi: 10.1080/07481187.2012.753555 pmid:24593008

31. Afonso SB, Minayo MC. Relationships between oncohematopediatrics, mothers and children in communicating bad news. Cien Saude Colet 2017;22:53-62. doi: 10.1590/1413-81232017221.14592016 pmid:28076529

32. Kassam A, Skiadaresis J, Alexander S, et al. Differences in end-of-life communication for children with advanced cancer who were referred to a palliative care team. Pediatr Blood Cancer 2015;62:1409-13. doi: 10.1002/pbc.25530 pmid:25882665

33. Bally JM, Smith NR, Holtslander L, et al. A metasynthesis: Uncovering what is known about the experiences of families with children who have life-limiting and life-threatening illnesses. J Pediatr Nurs 2018;38:88-98. doi: 10.1016/j.pedn.2017.11.004 pmid:29357986

34. Barry MJ, Edgman-Levitan S. Shared decision making—the pinnacle of patient-centered care. N Engl J Med 2012;366:780-1. doi:10.1056/NEJMp1109283 pmid:22375967

35. Zaal-Schuller IH, Willems DL, Ewals FVPM, et al. How parents and physicians experience end-of-life decision-making for children with profound intellectual and multiple disabilities. Res Dev Disabil 2016;59:283-93. doi:10.1016/j.ridd.2016.09.012 pmid:27665411

36. Badarau DO, Ruhe K, Kühne T, et al. Decision making in pediatric oncology: Views of parents and physicians in two European countries. AJOB Empir Bioeth. 2017;8:21-31. doi:10.1080/23294515.2016.1234519 pmid:28949870
37. Kane HL, Halpern MT, Squiers LB, et al. Implementing and evaluating shared decision making in oncology practice. CA Cancer J Clin 2014;64:377–88. doi:10.3322/caac.21245 pmid:25200391
38. Paediatric Association of the Netherlands (NVK). Richtlijn Palliatieve Zorg voor Kinderen [Guidelines on Palliative Care for Children]. 2018 [cited 2019 Oct 08]. Available from: http://richtlijnendatabase.nl/richtlijn/palliatieve_zorg_voor_kinderen
39. Beckstrand RL, Rawle NL, Callister L, et al. Pediatric nurses' perceptions of obstacles and supportive behaviors in end-of-life care. Am J Crit Care 2010;19:543-52. doi:10.4037/ajcc2009497 pmid:20026650
40. Van der Heide A, Onwuteaka-Philipsen BD, Rurup ML, et al. End-of-life practices in the Netherlands under the Euthanasia Act. N Engl J Med 2007;356:1957-65. doi: 10.1056/NEJMsa071143 pmid:17494928
41. Verhagen E, Sauer PJ. The Groningen protocol—euthanasia in severely ill newborns. N Engl J Med 2005;352:959-62. doi: 10.1056/NEJMp058026 pmid:15758003
42. Paediatric Association of the Netherlands (NVK). Standpunt NVK Levensbeëindiging kinderen 1-12 jaar: een pleidooi voor onderzoek. 2015. [cited 2019 Oct 08]. Available from: https://www.rijksoverheid.nl/documenten/publicaties/2016/04/22/standpunt-nvk-levensbeeindiging-kinderen-1-12-jaar-een-pleidooi-voor-onderzoek
43. Government of the Netherlands. Kamerbrief met reactie op NVK standpunt levensbeëindiging kinderen. 2016. [cited 2019 Oct 08]. Available from: https://www.rijksoverheid.nl/documenten/kamerstukken/2016/04/22/kamerbrief-met-reactie-op-nvk-standpunt-levensbeeindiging-kinderen
44. Rushton C. Integrating ethics and palliative care in pediatrics. Am J Nurs 2004;104:54–63. doi: 10.1097/00000446-200404000-00019 pmid:15171116
45. Hilhorst M, Deugden van de arts. In: Sanderse W, Kole J, eds. Karakter, Deugden voor professionals. Leusden: ISVW uitgevers 2018:103-12
46. Cheng Y, Lin CP, Liu HL, et al.Expertise modulates the perception of pain in others. Curr Biol. 2007;17:1708-13. doi:10.1016/j.cub.2007.09.020 pmid:17900903
47. Haque OS, Waytz A. Dehumanization in medicine: Causes, solutions, and functions. Perspect Psychol Sci. 2012;7:176-86. doi: 10.1177/1745691611429706 pmid:26168442
48. Haslam N. Humanising medical practice: the role of empathy. Med j Aust. 2007;187:381-2. doi: 10.5694/j.1326-5377.2007.tb01305.x pmid:17907998

For peer review only

Consolidated criteria for reporting qualitative studies (COREQ): 32-item checklist

Developed from:
Tong A, Sainsbury P, Craig J. Consolidated criteria for reporting qualitative research (COREQ): a 32-item checklist for interviews and focus groups. *International Journal for Quality in Health Care*. 2007. Volume 19, Number 6: pp. 349 – 357

YOU MUST PROVIDE A RESPONSE FOR ALL ITEMS. ENTER N/A IF NOT APPLICABLE

No. Item	Guide questions/description	Reported on Page #
Domain 1: Research team and reflexivity		
Personal Characteristics		
1. Interviewer/facilitator	Which author/s conducted the inter view or focus group?	First author Methods, p. 5
2. Credentials	What were the researcher's credentials? E.g. PhD, MD	MA Methods, p.5
3. Occupation	What was their occupation at the time of the study?	PhD student Methods, p.5
4. Gender	Was the researcher male or female?	Female Methods, p.5
5. Experience and training	What experience or training did the researcher have?	training Methods, p.5
Relationship with participants		
6. Relationship established	Was a relationship established prior to study commencement?	none Methods, p.5
7. Participant knowledge of the interviewer	What did the participants know about the researcher? e.g. personal goals, reasons for doing the research	Nothing Methods, p.5
8. Interviewer characteristics	What characteristics were reported about the inter viewer/facilitator? e.g. Bias, assumptions, reasons and interests in the research topic	None Methods, p.5
Domain 2: study design		
Theoretical framework		
9. Methodological orientation and Theory	What methodological orientation was stated to underpin the study? e.g. grounded theory, discourse analysis, ethnography, phenomenology, content analysis	Thematic analysis using constant comparative method. Methods, p.6
Participant selection		
10. Sampling	How were participants selected? e.g. purposive, convenience, consecutive, snowball	Maximum variety Methods, p.5
11. Method of approach	How were participants approached? e.g. face-to-face, telephone, mail, email	Various. See Methods, p.5

For peer review only

12. Sample size	How many participants were in the study?	64 parents of 44 children.<br><br>Results, see p.6
13. Non-participation	How many people refused to participate or dropped out? Reasons?	3, Methods. p.5
Setting		
14. Setting of data collection	Where was the data collected? e.g. home, clinic, workplace	Methods, interviews. p. 5
15. Presence of non-participants	Was anyone else present besides the participants and researchers?	Some parents were interviewed together with their spouse.<br>p.5
16. Description of sample	What are the important characteristics of the sample? e.g. demographic data, date	See Results, table 1 and 2. (p.6, p.7)
Data collection		
17. Interview guide	Were questions, prompts, guides provided by the authors? Was it pilot tested?	Methods, interviews see p.2
18. Repeat interviews	Were repeat inter views carried out? If yes, how many?	No.<br>Methods, p.5
19. Audio/visual recording	Did the research use audio or visual recording to collect the data?	Audio records<br><br>Methods, p.5
20. Field notes	Were field notes made during and/or after the inter view or focus group?	N/A
21. Duration	What was the duration of the inter views or focus group?	On average 2 hours.<br><br>Methods, p.5
22. Data saturation	Was data saturation discussed?	Yes<br>Methods, p.5
23. Transcripts returned	Were transcripts returned to participants for comment and/or correction?	Yes,<br>Methods. p.5
Domain 3: analysis and findings		
Data analysis		
24. Number of data coders	How many data coders coded the data?	Two main coders, checked by all 5 authors.<br>Methods see p.5
25. Description of the coding tree	Did authors provide a description of the coding tree?	See results, p.6. The coding tree can be added to the manuscript if needed.
26. Derivation of themes	Were themes identified in advance or derived from the data?	From the data.<br>See methods. p.5
27. Software	What software, if applicable, was used to	Atlas.ti,

For peer review only

	manage the data?	Methods, data analysis, see p.5
28. Participant checking	Did participants provide feedback on the findings?	By means of an advisory board.<br><br>Methods, p.6
Reporting		
29. Quotations presented	Were participant quotations presented to illustrate the themes/findings? Was each quotation identified? e.g. participant number	Yes, See results.
30. Data and findings consistent	Was there consistency between the data presented and the findings?	See results and discussion
31. Clarity of major themes	Were major themes clearly presented in the findings?	See Results
32. Clarity of minor themes	Is there a description of diverse cases or discussion of minor themes?	See results.

For peer review only

BMJ Open

Barriers in Care for Children With Life-Threatening Conditions: a Qualitative Interview Study in he Netherlands

Journal:	*BMJ Open*
Manuscript ID	bmjopen-2019-035863.R1
Article Type:	Original research
Date Submitted by the Author:	03-Mar-2020

Complete List of Authors:	Brouwer, Marije; Universitair Medisch Centrum Groningen, pediatrics<br>Maeckelberghe, Els; Universitair Medisch Centrum Groningen, Institute for Medical Education FC 40<br>van der Heide, Agnes; Erasmus MC, University Medical Center Rotterdam, Clinical Microbiology<br>Hein, Irma; Academic Medical Center: Amsterdam, psychiatry<br>Verhagen, Eduard; University Medical Center Groningen, University of Groningen, Dept of Pediatrics

**Primary Subject Heading**:	Paediatrics
Secondary Subject Heading:	Palliative care, Ethics
Keywords:	PALLIATIVE CARE, MEDICAL ETHICS, Paediatric palliative care < PALLIATIVE CARE

SCHOLARONE™
Manuscripts

I, the Submitting Author has the right to grant and does grant on behalf of all authors of the Work (as defined in the below author licence), an exclusive licence and/or a non-exclusive licence for contributions from authors who are: i) UK Crown employees; ii) where BMJ has agreed a CC-BY licence shall apply, and/or iii) in accordance with the terms applicable for US Federal Government officers or employees acting as part of their official duties; on a worldwide, perpetual, irrevocable, royalty-free basis to BMJ Publishing Group Ltd ("BMJ") its licensees and where the relevant Journal is co-owned by BMJ to the co-owners of the Journal, to publish the Work in this journal and any other BMJ products and to exploit all rights, as set out in our licence.

The Submitting Author accepts and understands that any supply made under these terms is made by BMJ to the Submitting Author unless you are acting as an employee on behalf of your employer or a postgraduate student of an affiliated institution which is paying any applicable article publishing charge ("APC") for Open Access articles. Where the Submitting Author wishes to make the Work available on an Open Access basis (and intends to pay the relevant APC), the terms of reuse of such Open Access shall be governed by a Creative Commons licence – details of these licences and which Creative Commons licence will apply to this Work are set out in our licence referred to above.

Other than as permitted in any relevant BMJ Author's Self Archiving Policies, I confirm this Work has not been accepted for publication elsewhere, is not being considered for publication elsewhere and does not duplicate material already published. I confirm all authors consent to publication of this Work and authorise the granting of this licence.

For peer review only

Barriers in Care for Children With Life-Threatening Conditions: a Qualitative Interview Study in he Netherlands

MARIJE BROUWER, ELS MAECKELBERGHE, AGNES VAN DER HEIDE, IRMA HEIN, and EDUARD VERHAGEN

Marije A. Brouwer, MA
m.a.brouwer@umcg.nl
Department of paediatrics, University of Groningen, University Medical Center Groningen, Groningen, the Netherlands

dr. Els L.M. Maeckelberghe
e.l.m.maeckelberghe@umcg.nl
Institute for Medical Education, University of Groningen, University Medical Center Groningen, Groningen, the Netherlands

prof. Agnes van der Heide
a.vanderheide@erasmucmc.nl
Department of Public Health, Erasmus medical Center, Rotterdam, the Netherlands

dr. Irma M. Hein
i.hein@debascule.com
Department of psychiatry, Academic Medical Center, Amsterdam, the Netherlands

prof. A.A.E. Eduard Verhagen
a.a.e.verhagen@umcg.nl
Department of paediatrics, University of Groningen, University Medical Center Groningen, Groningen, the Netherlands

Address correspondence to:
Marije Aafke Brouwer
University Medical Center Groningen, Department of Pediatrics,
tnv A.J.Heinstra/M.A.Brouwer.
Antwoordnummer 333, 9700 VB Groningen
mail: m.a.brouwer@umcg.nl
phone: 0031-621102049

Word count (excluding abstract, references, and quotes): 3943

For peer review only

Abstract.

Objective: To identify barriers, as perceived by parents, to good care for children with life-threatening conditions.

Design: In a nationwide qualitative study, we held in-depth interviews regarding end-of-life care with parents of children (aged one to 12 years) who were living with a life-threatening illness or who had died after a medical trajectory (a maximum of five years after the death of the child). Sampling was aimed at obtaining maximum variety for a number of factors. The interviews were transcribed and analysed.

Setting: The Netherlands.

Participants: 64 parents of 44 children.

Results: Parents identified six categories of difficulties that create barriers in care for children with a life-threatening condition. First, parents wished for more empathetic and open communication about the illness and prognosis. Second, organizational barriers create bureaucratic obstacles and a lack of continuity of care. Third, parents wished for more involvement in decision-making. Fourth, parents wished they had more support from the health care team on end-of-life decision-making. Fifth, parents experienced a lack of attention for the family during the illness and after the death of their child. Sixth, parents experienced an overemphasis on symptom-treatment and lack of attention for their child as a person.

Conclusions: The barriers as perceived by parents focused almost without exception on non-medical aspects: patient-doctor relationships, communication, decision-making, including end-of-life decision-making, and organization. The perceived barriers indicate that care for children with a life-threatening condition focuses too much on symptoms and not enough on the human beings behind these symptoms.

For peer review only

Strengths and limitations.

Strengths:

- The variety: Parents from children with various illnesses, ages and prognosis, receiving care from various hospitals were included.
- The size: 64 participating parents from 44 children were interviewed, and over 110 hours of qualitative data on audio were collected.

Limitations:

- The study focuses on young children (aged one to 12 years old) and the results cannot be generalized to adolescents or neonates.

For peer review only - http://bmjopen.bmj.com/site/about/guidelines.xhtml

- The success in achieving variation in the cultural and religious background of participants with different cultural and religious backgrounds variation was limited.
- The study reports only the barriers experiences by parents.

For peer review only

For peer review only - http://bmjopen.bmj.com/site/about/guidelines.xhtml

Introduction.

Parents and physicians caring for children with life-threatening conditions face multiple challenges in providing the best care for children. Care for children whose futures and chances of survival are uncertain is often a complex trajectory, where curative and palliative care entwines.[1, 2] Curative care goals – to cure/prolong life – and palliative care goals – to relieve suffering – sometimes collide, complicating care. Care for these children is further complicated because decisions that may limit the life-expectancy of a child (including end-of-life decisions) are considered part of palliative care [3, 4]

Life-threatening conditions are defined as conditions for which curative treatment may be feasible, but for which this treatment could also fail, leading to a possibility of dying.[5] Care for children with life-threatening conditions can include aspects of both curative and palliative care at the same time.[2] Healthcare providers are not always confident about their skills in providing palliative care.[6] The main reported barriers in paediatric palliative care as experienced by healthcare providers are communication, the uncertainty of the prognosis, time constraints and lack of education.[7-11] Studies on barriers experienced by parents show that communication is a major barrier for them.[12-17] Other frequently mentioned barriers are a lack of care for the family[12-13] and bereavement care.[17]

But knowledge of parents' experiences of barriers to good care is limited in at least two ways. First, it focuses mainly on specific health conditions, particularly on oncology,[14,15] and second, it focuses mainly on children receiving palliative care, often with a certain, lethal prognosis.[12-13,16-17] However, not all children who have life-threatening conditions receive palliative care, or are diagnosed with a lethal prognosis.[18-20]

To provide insight into barriers to care and decision-making for children living with life-threatening conditions, we conducted a qualitative study throughout the Netherlands. We interviewed parents who had experience in caring for a child with a life-threatening condition to identify what they regarded as barriers to both good care and good decision-making.

Methods.

For peer review only - http://bmjopen.bmj.com/site/about/guidelines.xhtml

In a large-scale, nationwide qualitative study we interviewed parents of children, either living with a life-threatening or terminal condition, or who had died after a medical trajectory (a maximum of five years before the date of the interview). Children were aged between one and 12 years old and had a variety of life-threatening conditions. The study was reviewed and deemed exempt by the medical ethical committee of the University Medical Center Groningen.

Recruitment.

Participants were recruited between November 2016 and October 2018. To recruit participants, all Dutch paediatricians received an invitation from the Paediatric Association of the Netherlands (NVK) to invite potential parents to join the research.[21] A website with information about the study was created to inform potential participants.[22] Parent support groups in oncology, cardiology, metabolic and neuromuscular diseases and palliative care helped to reach potential participants through their online platforms. To ensure maximum variety in ethnic background we recruited through physicians with expertise in treating patients with different cultural backgrounds. Sampling was aimed at obtaining maximum variety in terms of health condition, age, cultural background, parental level of education and place of residence. Three participants dropped out due to scheduling difficulties. Recruitment continued until thematic saturation and saturation of variety in participants was achieved.

Interviews.

Parents were interviewed in face-to-face in-depth interviews, usually held at the parent's place of residence. The first author (MA, PhD student, female) conducted the interviews after following formal training. The participants signed an informed consent form before the interview. Parents did not know the interviewer before the study. All participants were given the choice to conduct the interview alone or together with a co-parent. A topic guide had been developed as an interview guide. The interview covered the following themes: course of the disease, symptoms, suffering, care and decision-making and end of life. The topic guide is added as a supplementary file to the manuscript. The interviews were recorded on audio and subsequently transcribed verbatim. All interviews were anonymized during transcription. The average duration of the interviews was two hours. Participants were given the opportunity to revise their transcribed interviews.

Analysis.

For peer review only

For this article, we selected quotes concerning perceived barriers in care. For reasons of practicality in handling the approximately 2,500 pages of transcripts, the first author gathered all codes on barriers in care in parent interviews, which were subsequently read by all authors to become familiarized with the content. The first author further focused on parents' perceptions of barriers in care in the selection of interview fragments, and this was reviewed by all authors. The research team (the authors of this paper) consisted of a PhD student, an ethicist, a professor in end-of-life care, and a child psychiatrist and a physician/professor in paediatric palliative care.

A primary thematic analysis, aiming at a qualitative description of barriers as perceived by parents,[23] was performed using the constant comparative approach.[24] Coding was performed by the first two authors and reviewed by all authors. Differences were settled by discussion until consensus was achieved.

Patient and Public Involvement.

The focus of this study is on parent experiences and preferences. During the study, biannual meetings were held with an advisory board (9 members) of parents, physicians and researchers who provided feedback on the findings. They were offered remuneration for their efforts. They provided substantial input on the study design, recruitment, analysis and the reporting of results of the study. All participants were given the opportunity to check and revise their manuscript, and are regularly updated on the outcomes of the study by e-mail.

Results.

Participants. We interviewed 64 parents of 44 children. Our participants came from all over the Netherlands. All children received, or had received, care in at least one university medical centre, often combined with care in one or more local hospitals. All eight university hospitals in the Netherlands, as well as over 20 local hospitals, were represented in the study. Characteristics of children and parents are shown in Tables 1,2 and in Figure 1.

For peer review only

Table 1. Characteristics of children (n=44)

Status, deceased	27 (61.4%)
Gender, male	20 (45.5%)
Age (years)	
1-3	15 (34.0%)
4-6	8 (18.2%)
7-9	9 (20.5%)
10-12	12 (27.3%)
Diagnosis	
Malignancies	18 (40.9%)
Neurological/metabolic	17 (38.6%)
Cardiovascular	4 (9.1%)
CNS	3 (6.8%)
Other	1 (2.2%)
Undiagnosed	1 (2.2%)

For peer review only

Table 2. Characteristics of parents (n=64 interviewed parents, 42 couples)

Relationship status	
Married/Together	34 (81.0%)
Level of education, mothers (N=42)[1]	
Low-level education (< 4 years)	1 (2.4%)
Mid-level education (practical, 4 years)	15 (35.7%)
Higher education (vocational, 4 years),	14 (33.3%)
Academic education (University, 4-6 years)	12 (28.6%)
Level of education, fathers (N=42)[1]	
Low-level education (< 4 years)	4 (9.5%)
Mid-level education (practical, 4 years)	15 (35.7%)
Higher education (vocational, 4 years),	14 (33.3%)
Academic education (University, 4-6 years)	9 (21.4%)
Ethnicity of parents, according to the participants (N=42)	
Dutch	36 (85.7%)
Mixed	6 (14.3%)
Religious/ spiritual beliefs (N=64)	
None	39 (60.9%)
Christian	19 (29.7%)
Other	6 (9.4%)
Family composition	
1 child	8 (19.0%)
2 children	22 (52.4%)
3 children	10 (23.8%)
4 or more children	2 (4.8%)

[1.] We considered the educational level of both parents to be possibly of influence on the care and decisions of the child. Therefore the educational level of both parents is represented, even if one of them was not interviewed.

only

Experienced barriers. We identified six major barriers in care for children with life-threatening conditions: We summarized the barriers in Table 3.

For peer review only - http://bmjopen.bmj.com/site/about/guidelines.xhtml

Table 3. Barriers in paediatric palliative care, as experienced by parents.

**Barrier 1. Parents wished for more empathic and open communication about the illness and prognosis.**
1a. Absence of conversations about the future and possible prognosis.
1b. Communication lacks empathy.
**Barrier 2: Organizational barriers create bureaucratic obstacles and a lack of continuity of care.**
2a. Lack of continuity in care threatens good care
2b. Healthcare structures create bureaucratic obstacles.
**Barrier 3. Parents wished for more involvement in decision-making**
**Barrier 4. Parents wished they had more support from the health care team on end-of-life decision making**
**Barrier 5. Parents experienced a lack of attention for the family during the illness and after the death of their child.**
5a. Impact on parents and siblings is neglected in care.
5b. Parents experience a lack of bereavement care.
**Barrier 6. Parents experience an overemphasis on symptom-treatment and lack of attention for their child as a person.**
6a. Lack of attention for how a child might experience treatment and illness.
6b. Failure to evaluate symptoms in the context of the child's illness as a whole.
6c. Overemphasizing the patients role as a sick person instead of a human being .

Barrier 1: Parents wished for more empathetic and open communication about the illness and prognosis

Parents univocally felt that the wish to receive complete and open information, and the absence thereof, was a significant barrier to good care. Most experiences regarding communication involved either a perceived lack of openness about the prognosis or a lack of empathy in communication.

1a. Absence of conversations about the future and possible prognosis.

Telling a parent that their child may not survive might seem like a message that parents would not like to receive, but parents (both bereaved parents and non-bereaved parents) stressed that they had wished for open conversations about their child's future. Not talking about the prognosis creates uncertainty and makes the topic a taboo. They underlined the importance to openly talk about this, especially when the prognosis is uncertain.

For peer review only - http://bmjopen.bmj.com/site/about/guidelines.xhtml

> (F02): 'In the first few years, you still have the hope that things might change, but after a few years, you realize that almost all of that hope has gone. It would be great if physicians would take you by the hand and say: "We have to accept that this is what it is, and that things are probably not going to get better." And ask you as a parent: "What are your thoughts on that? Would you, despite that prognosis, like to keep on trying new treatments? Or do you think it is better not to do that?"'

Parents specifically needed openness about the prognosis to discuss the possibility of transition between different levels of concurrent life-prolonging treatments and comfort care, or to make end-of-life decisions. In a few cases, the topic of dying was only mentioned a day before the death of their child.

1b. Communication lacks empathy. Parents experienced a lack of empathetic communication. They felt that the friction between healthcare professionals doing their daily jobs and families going through a once-in-a-lifetime event was characteristic for the lack of sympathy that they experienced.

> (M27): '[the lady who came to explain the procedure of palliative sedation] was so insensitive. She just explained all the technicalities, with a broad smile. And I remember thinking: "It's not like I am buying bread from you!" (…) It was as if she thought: "let's get this over with", as if there was not an entire life, an entire family, an entire world affected by the procedure that she was explaining.'

But experienced barriers in communication were not limited to interactions with healthcare providers or hospital settings.

> (F16): 'Before we had even held the funeral, we received a letter from the local authority asking us to return our special parking permits as soon as possible. Just like that, without any condolences. Bizarre.'

Barrier 2: Organizational barriers create bureaucratic obstacles and a lack of continuity of care.

With regard to organization, two themes emerged: continuity of care and bureaucracy.

For peer review only

2a. Lack of continuity in care threatens quality. Parents described having to deal with many different care providers, either individual professionals or organizations, which made it difficult to guarantee continuity of care. Agreements made with one provider did not always dovetail well with the care that they received from other care providers.

> (F03): 'There was a physician on duty, and when we updated him on the 'do not resuscitate' agreements that we had made with our regular physician, he said: "No, I'm not going to do that. It is my shift, and my responsibility, and I'm not going to do that." You take all this time to make decisions with your doctor, and then another doctor, who happens to be on duty and doesn't know your child or the file, who has never had a conversation with you, simply states: "I'm responsible, so I determine what happens."'

2b. Healthcare structures create bureaucratic obstacles.. Parents particularly experienced bureaucratic barriers to good care in situations where they had to deal with a multitude of healthcare-related organizations.

> (M31) 'When the neurologist prescribes some sort of medicine, he has to send it by fax to the pharmacist, he can't send it by email. And if there is even one comma in the wrong place, the pharmacist won't prescribe the medicine that my son needs.'

Several parents expressed the need for a case manager, who could bridge the gaps between parents, different healthcare providers and financers. Notably, this barrier was also mentioned by parents who had received palliative care from teams that already worked with case managers. While more and more paediatric palliative teams in the Netherlands are appointing case managers to families dealing with a life-threatening illness, the question arises as to how they can be used in the most effective manner.

Barrier 3: Parents wished for more involvement in decision-making

The third barrier concerned participation in decision-making. There was a lot of variation regarding the involvement of parents in decision-making. Parents who had been involved in decision-making, appreciated it.

> (M15): '[when we had to decide whether to go for radiotherapy or not] our doctor said: "Whatever your decision is, there is no wrong answer, and I will stand by your choice."'

For peer review only - http://bmjopen.bmj.com/site/about/guidelines.xhtml

Other parents described that they were not as involved in decision-making as they would have liked, both for decisions made during curative treatment and for those made in the terminal phase. One parent described that the decision to withdraw life-sustaining treatment for their child was made by a neurologist who had never spoken to them.

> (M27): 'The neurologist never spoke to us about his motivations to [withdraw treatment] and start the death of our son. He came in, made the observation right in front of us, but only spoke in private to our paediatrician, and left.'

All participants stressed the importance of involving parents in the decision-making process, stating that the decisions included considerations about the suffering, quality of life and daily life of the child, something in which the parents' expertise was needed. The only exception parents mentioned to being involved, were decisions that parents saw as 'merely medical' information, such as technicalities about medication or treatment.

Barrier 4. Parents wished they had more support from the health care team at end-of-life decision-making

Parents mentioned discussions about the possibility of transition between different levels of concurrent life-prolonging treatments and comfort care, or to make end-of-life decisions, as major themes in the care for their child. Parents sometimes had doubts about the appropriateness of continued treatment or felt that it was wrong that parents themselves had to initiate a conversation about end-of-life decision-making.

Additionally, several parents expressed a wish for more legal possibilities for active life-ending in order to relieve their child's suffering and grant their child a dignified end of life. In the Netherlands, active life-ending for children – sometimes referred to as paediatric euthanasia – is not legal for children aged between one and 12 years old.[25] Withholding or withdrawing treatment and palliative sedation are considered permitted medical practices.[26]

For the majority of the children in the study, end-of-life decisions were made, ranging from withholding or withdrawing life-prolonging or life-sustaining treatment to palliative sedation. Nevertheless, in several cases, parents still felt the need for an option to make end-of-life decisions. In most cases, their need came from a situation in which the condition of their child

For peer review only - http://bmjopen.bmj.com/site/about/guidelines.xhtml

was physiologically stable and the physicians could not justify the withdrawal of treatment or palliative sedation.

> (M02) 'Physicians told us that if they had to [give her] so much morphine to ease her pain that the morphine itself would be the cause of her death, it would be considered active life-ending, which is not allowed for children who cannot ask for it themselves. But we would want that for her, because we have the feeling that the only thing that can bring her comfort and peace (emotional) will be death.'

In other situations, parents felt a need for active life-ending because the existing options were unable to grant their child a dignified end-of-life.

> (M07): 'If we all know that the palliative sedation is going to result in his death, why does he have to go through these final days of suffering? Why does it have to be so... undignified?'

Barrier 5. Parents experienced a lack of attention for the family during the illness and after the death of their child.

A life-threatening illness does not only affect children, but also their families. Parents felt that they and their families were not always supported during and after the illness of their child. Siblings in particular were often overlooked.

> (M20): 'As a parent you get a little support, but for brothers and sisters there is very little to absolutely no help at all. They have to figure it out for themselves, it seems as though they are forgotten.'

While support for the family is one of the aims of paediatric palliative care,[26] and all Dutch hospitals have psychologists, social workers and/or palliative care teams to provide this, it seems that this support is not always offered to parents or did not function as well as parents had hoped. However, for parents, the need was not so much about offering psychological support as it was about empathy in their contact with the healthcare providers that they came across.

> (M05) 'What they don't always realize is that the hospital also becomes your social world; the contact with doctors, the chats with nurses.'

For peer review only - http://bmjopen.bmj.com/site/about/guidelines.xhtml

Barrier 6. Parents experience an overemphasis on symptom-treatment and lack of attention for their child as a person.

The final barrier expressed by parents was a wish for healthcare providers to understand the fact that patients are persons, not merely carriers of certain symptoms. They explained that healthcare professionals often failed to evaluate the impact of the condition or the medical interventions on the child's daily life. This barrier had three different aspects: 1) a lack of attention for how a child might understand and cope with treatment and illness, 2) failure to see the complexity of the child as a whole and, finally, 3) overemphasizing the child as a patient instead of as a whole person.

6a. Lack of attention for how a child might experience treatment and illness.

One specific characteristic of paediatric care is the attention given to how children understand and cope with their condition and the associated treatment. Parents underlined that this is important both for 'bigger questions' and for simple medical interventions. Parents mentioned the comprehension of the child as a vital aspect in the child's ability to cope with illness and treatment.

> (M04) 'Hospitals are busy with production. So, a nurse comes in, and basically jumps on the child to take his blood pressure, or give an injection, without paying attention to what it does to him (…) And I often wonder: How hard can it be to ask yourself beforehand how to approach this individual child, so that he understands the interventions?'

Parents saw taking the time to explain to children what is happening them (instead of performing treatment on them without explanation) as an important aspect of taking children seriously as people.

6b Failure to evaluate symptoms in the context of the child's illness as a whole.

Children with life-threatening conditions can receive treatment from various specialists. Parents described how specialists sometimes failed to look beyond the specific symptoms that they were treating and see the child and the complexity of their condition as a whole.

> (M29): 'We had an incident because my son had fallen, and the dental surgeon took one look at his teeth and said: "Those teeth have got to go." And I said: "But what then? He already has

For peer review only - http://bmjopen.bmj.com/site/about/guidelines.xhtml

problems swallowing." And he said casually: "He'll need a feeding tube." Not caring what it would mean to him, and our lives, to make that decision. And I don't think he even saw the boy who was sitting there, who I love so much, and who I am so worried about.'

6c. Overemphasizing the patients role as a sick person instead of a human being.

The final barrier identified was the overemphasis of the child as a patient, at the cost of the child as a person. Parents described how medical treatment sometimes overemphasized the child as a patient and that there was little attention for the child as a person, impeding the child's quality of life. Parents stressed that especially for ill children keeping up a normal life became very important: going to school, not standing out, feeling like an ordinary child. Daily activities that did not focus on the child's illness, but on the child as an ordinary person, became cherished. As this mother remembers:

(M14): 'It was the highlight of his day, when his teacher would come; solving math problems (laughs).'

This barrier also influenced the readiness of a child to communicate about their illness. Children did not always want to talk about the illness, a few even forbidding their parents to talk about it with others. Seeing the child too much as a patient, either by focussing merely on the child's illness, or by trying to make them feel special, might deprive children of living a normal life.

Both barriers five and six refer to what parents described as their wish for a more human-centred approach to care, which also implied an empathic connection between professionals and parents/patients. According to the parents, some physicians perceived this attitude as unprofessional:

(M20): 'One physician had a big impact on me. We had to go home, and she said goodbye to my son. She started crying and apologized: "I'm so sorry, I'm being very unprofessional."'

For parents, however, this kind of connection was much appreciated and in fact seen as a sign of professionalism.

For peer review only - http://bmjopen.bmj.com/site/about/guidelines.xhtml

(M20, continued): 'And I said: "No, right now, you are human." I wonder if she knows that of all those doctors, she is the only one that crosses my mind frequently. Because she cared.'

Discussion.

Main findings. This qualitative study gives insight into parents' perceived barriers in care for children with life-threatening conditions. The barriers experienced by parents focused almost without exception on the non-technical skills in medicine: attention for children, empathy, communication, decision-making and organization. The absence of perceived barriers on the execution of symptomatic care is notable.

With respect to the first barrier, communication, other studies report communication as a barrier for both physicians [7-8,11,27-31] and parents.[12-16,32-34] Our results underline the importance of openness as a requirement for decision-making, and adds that communication is not limited to hospital settings. A possible future development for paediatric palliative care is to extend communication training to all professionals dealing with children with life-threatening conditions, not merely hospital workers.

To reduce organizational barriers, parents suggested introducing case managers that might help parents and children with bridging the gaps between different healthcare professionals and in dealing with bureaucratic difficulties. Over the past year, several paediatric palliative care teams in the Netherlands have introduced such case managers, with the aim of trying to reduce this barrier. However, it is worth mentioning that in current practice, palliative care is predominantly limited to terminally ill children, and consequently, these palliative care services also remain limited to the terminal phase. However, issues with the continuity of care and with bureaucracy may arise long before this phase.

With regard to the third barrier, decision-making, the involvement of parents in decision-making varied. Previous studies also show that parents are not always involved in decision-making.[35-36] However, while these studies illustrate that some parents do not wish to be involved in decision-making,[37-38] all parents in our study expressed the importance of being involved in decision-making, with the only exception of decisions that involved merely medical knowledge. These differences may be explained by the different timing of decision-making processes (critical care versus longer term care). It is also possible that the

For peer review only - http://bmjopen.bmj.com/site/about/guidelines.xhtml

understanding of parents on what it means to be involved differs between studies (for example, being heard versus being fully responsible).

Shared decision-making has been an important movement in both paediatrics and medicine in general over the last decade.[39-40] Dutch regulations underline the importance of making parents, and – if possible – even children themselves, part of the decision-making process.[41] The fact that parents often feel excluded may be an incentive to further investigate why parents are not always included, and what is needed to achieve this.

The perceived need for end-of-life decisions is a barrier that, to our knowledge, has not previously been mentioned as a barrier in studies researching barriers in care for children with life-threatening conditions. In contrast, other studies mention parents' discomfort with making end-of-life decisions such as withholding or withdrawing treatment.[42] Although the Netherlands is known for having a legal regulation of euthanasia and end-of-life decision-making in adults and newborns,[25,43-44] the current regulations for children aged between one and 12 years old are similar to those in most other countries. Withholding and withdrawing treatment and palliative sedation are permitted, but active life-ending (euthanasia) is not. Following the Belgian legalization of euthanasia for competent minors, there has been a debate in the Netherlands about whether regulations on euthanasia should be broadened to allow active life-ending for children younger than 12 in cases of unbearable and hopeless suffering.[45-46]

Parents perceived a lack of care for the family. Family care is one of the goals of palliative care[8] and is arguably a service that all parents and siblings of the child should receive.[18] Previous studies highlight the importance of family support, including bereavement care.[12-13,17]

Previous studies have underlined that care for young children includes attention for a child's development.[18,47] Our study adds that this does not only mean seeing the child as a whole, with both physical, social and emotional aspects, but that it also means seeing the child as a *person*. From the perspective of parents, care for children with life-threatening conditions focuses too much on symptoms and not enough on the human beings behind the symptoms. This raises the question of whether such an attitude should be part of medical professionalism.

For peer review only - http://bmjopen.bmj.com/site/about/guidelines.xhtml

Medical professionalism: dehumanization?

Medicine and emotional intimacy seem to have a difficult relationship. Can a professional working in paediatric palliative medicine be expected to build an empathic, intimate relationship with all of their patients? Some authors argue that what makes a good doctor is, in essence, expertise and knowledge, not empathy; that good doctor may be stand-offish or surly, as long as they treat a symptom adequately.[48] Intimacy is a tool needed to detect the needs of a patient, but should be used very carefully, otherwise a doctor might 'lose himself".[48-50] Nevertheless, empathy benefits patients.[51] The view of detachedness as a professional quality is often – although not always explicitly – reiterated throughout medicine. There is a tension between this viewpoint of professional detachedness and that of empathic intimacy, or human-to-human contact, that parents ask for.

What parents seem to miss in current practise is the other connotation of the word 'care': to care about. We cannot expect physicians or nurses to become emotionally attached to every patient that they meet, but this is not what parents seem to miss in current care. What they miss in care is not the lack of showing emotion, but the affirmation that all parties – children, family and physicians – are in fact human and that the illness affects lives, not monitors. This attitude can be implemented into standard care for children with life-threatening conditions.

Medical detachedness versus attention for persons is not black or white, but rather a continuum, ranging from patient-physician relationships that focus on very specific symptoms, without much attention for the person who is experiencing it, towards recognizing the complexity of the child's overall health situation, and finally evaluating care in the context of a child who as a human being experiences its illness. In different forms of medicine different levels of detachedness, and attention for the person may be appropriate. A dental appointment, where the physician-patient relationship is short, and the treatment limited to a specific expertise, calls for a different kind of professionalism than paediatric palliative care, where its very goal implies attention for the person. By its goal –improving quality of life by relieving suffering–[2] paediatric palliative care has shifted its focus from the illness and symptom towards the patient. The experienced barriers indicate that this shift is possibly insufficiently translated into care. Perhaps it is time to consider how we should evaluate professional detachedness in the light of the goals of palliative care. Above all, policies and

For peer review only - http://bmjopen.bmj.com/site/about/guidelines.xhtml

guidelines should not merely guide professionals on the technical aspects of symptom treatment, but aid them in topics of professional intimacy as well.

Strengths and limitations.

Our study has some limitations. First, our study focused on young children (aged one to 12 years old) and the results cannot be generalized to adolescents or neonates. Second, although we put considerable effort into recruiting participants with different cultural and religious backgrounds, the success in achieving this variation was limited. Finally, we did not include the perspectives of physicians, in order to solely present the barriers perceived by parents. Although the parents' own experiences are the experiences that they live by, and that represent their reality, they do not present a complete overview alone. In order to fully evaluate care for children with life-threatening conditions, the reality of the physicians, nurses and other involved parties should also be described.

The strengths of this study are the size of the study. We held in-depth interviews with 64 participating parents, and over 110 hours of qualitative data on audio were collected. This makes the study unique in its size. A second strength is the variety. Most existing research is limited either by medical diagnosis or hospital where care is administered. In this study we interviewed parents from children with various illnesses, in various stages of their illness, receiving care from various hospitals, providing a much needed broad perspective on care given to children living with life-threatening conditions.

Conclusion

This study explores the barriers that parents encounter during the process of caring for children with life-threatening conditions. The barriers experienced by parents in care for children with life-threatening conditions and uncertain futures are related to six major themes. First, parents wished for more empathetic and open communication about the illness and prognosis. Second, organizational barriers create bureaucratic obstacles and a lack of continuity of care. Third, parents wished for more involvement in decision-making. Fourth, parents wished they had more support from the health care team on end-of-life decision-making. Fifth, parents experienced a lack of attention for the family during the illness and after the death of their child. Finally, parents experience an overemphasis on symptom-treatment and lack of attention for their child as a person. The parents' wish to see the child as a person sheds new light on the relationship between medical professionalism and detachedness. We argue that in paediatric palliative care, the child behind the symptoms is

For peer review only - http://bmjopen.bmj.com/site/about/guidelines.xhtml

sometimes forgotten. Paediatric palliative care might suit the needs of parents and children better when it re-evaluates its current professional detachedness, and progresses towards a medical professionalism where not only symptoms, but also people are treated.

Acknowledgements.

The authors would like to thank the participants for sharing their stories, the members of the advisory group and Veerle van de Wetering for their help in shaping the study and heir help in interpreting the results, and Suzanna Miedema-Wilsterman for her efforts in recruiting participants for this study.

Contributorship statement

All authors are responsible for the reported research, and article and meet the ICMJE standards for authorship. All authors agree to be accountable for all aspects of the work in ensuring that questions related to the accuracy or integrity of any part of the work are appropriately investigated and resolved.

MB contributed to the study design, conducted the recruitment and interviews, performed the qualitative analysis and drafted the manuscript.

EM contributed to the study design, helped in recruiting participants, performed the qualitative analysis, and contributed to the writing of the manuscript.

AvdH contributed to the study design, checked the qualitative analysis, and contributed to the writing of the manuscript.

IH contributed to the study design, contributed to the study design, checked the qualitative analysis, and contributed to the writing of the manuscript.

EV contributed to the study design, helped in recruiting participants, checked the qualitative analysis, and contributed to the writing of the manuscript.

Competing interest statement

This work was supported by the Dutch Ministry of Health, Welfare and Sport. Grant number 324717 (560019968)]. The authors have no further conflict of interest or financial relations to disclose.

Ethics approval.

For peer review only

The study was reviewed and deemed exempt by the medical ethical committee of the University Medical Center Groningen. Number: METC2016. 223.

Figure legend/supplemental files.

Figure 1.

Topic guide.

References

1. Mack JW, Wolfe J. Early integration of pediatric palliative care: for some children, palliative care starts at diagnosis. Curr Opin Pediatr 2006;18:10-4. doi:10.1097/01.mop.0000193266.86129.47 pmid:16470155
2. World Health Organization. WHO definition of palliative care. [cited 2019 Oct 08]. Available from: https://www.who.int/cancer/palliative/definition/en/
3. Brouwer M, Maeckelberghe E, De Weerd W, et al. Quality of Living and Dying: Pediatric Palliative Care and End-of-Life Decisions in the Netherlands. Camb Q Healthc Ethics 2018;27:376-84. doi:10.1017/S0963180117000767 pmid:29845907
4. Paediatric Association of the Netherlands (NVK). Richtlijn Palliatieve Zorg voor Kinderen. 2018 [cited 2019 Oct 08]. Available from: http://richtlijnendatabase.nl/richtlijn/palliatieve_zorg_voor_kinderen
5. International Children's Palliative Care Network (ICPCN) What is children's palliative care? 2010. [cited 2019 Oct 08]. Available from: http://www.icpcn.org.uk/page.asp?section=0001000100080004§ionTitle=What+is+Children%92s+Palliative+Care%3F
6. Sheetz MJ, Bowman MA. Pediatric palliative care: an assessment of physicians' confidence in skills, desire for training, and willingness to refer for end-of-life care. American Journal of Hospice and Palliative Medicine 2008;25:100-5. doi:10.1177/1049909107312592 pmid:18445861
7. Davies B, Sehring SA, Partridge JC, et al. Barriers to palliative care for children: perceptions of pediatric health care providers. J. Pediatr 2008;121:282-8. doi:10.1542/peds.2006-3153 pmid:18245419
8. Beckstrand RL, Rawle NL, Callister L, et al. Pediatric nurses' perceptions of obstacles and supportive behaviors in end-of-life care. Am J Crit Care. 2010;19:543-52 doi:10.4037/ajcc2009497 pmid:20026650
9. Gowan D. End-of-life issues of children. Pediatr Transplant 2003;7:40-3. doi:10.1034/j.1399-3046.7.s3.5.x pmid:12603691
10. Bartel DA, Engler AJ, Natale JE, et al. Working with families of suddenly and critically ill children: physician experiences. Arch Pediatr Adolesc Med 2000;154:1127-33. doi:10.1001/archpedi.154.11.1127 pmid:11074855

For peer review only

11. Williams‐Reade J, Lamson AL, Knight SM, et al. Paediatric palliative care: A review of needs, obstacles and the future. J Nurs Manag 2015;23:4-14. doi:10.1111/jonm.12095 pmid:23944156
12. Davies B, Connaughty S. Pediatric end-of-life care: Lessons learned from parents. J Nurs Adm 2002;32:5-6. doi:10.1097/00005110-200201000-00001 pmid:11802640
13. Contro N, Larson J, Scofield S, et al. Family perspectives on the quality of pediatric palliative care. Arch Pediatr Adolesc Med 2002;156:14-9. doi:10.1001/archpedi.156.1.14 pmid:11772185
14. Tenniglo LJ, Loeffen EA, Kremer LC, et al. Patients' and parents' views regarding supportive care in childhood cancer. Support Care Cancer 2017;25:3151-60. doi:10.1007/s00520-017-3723-7 pmid:28456909
15. Nyborn JA, Olcese M, Nickerson T, et al. "Don't try to cover the sky with your hands": parents' experiences with prognosis communication about their children with advanced cancer. J Palliat Med 2016;19:626-31. doi:10.1089/jpm.2015.0472 pmid:27115314
16. Hsiao JL, Evan EE, Zeltzer LK. Parent and child perspectives on physician communication in pediatric palliative care. Palliat Support Care 2007;5:355-65. doi:10.1017/S1478951507000557 pmid:18044413
17. Widger K, Picot C. Parents' perceptions of the quality of pediatric and perinatal end-of-life care. Pediatr Nurs 2008;34;53-8.
18. Goldman A. ABC of palliative care: special problems of children. BMJ. 1998;316:49–5. doi: 10.1136/bmj.316.7124.49 pmid:9451269
19. Hynson JL, Gillis J, Collins JJ, et al. The dying child: how is care different? Med J Aust. 2003;179:20–2. doi:10.5694/j.1326-5377.2003.tb05571.x pmid:12964930
20. Mack JW, Wolfe J. Early integration of pediatric palliative care: for some children, palliative care starts at diagnosis. Curr Opin Pediatr 2006;18:10-4. doi:10.1097/01.mop.0000193266.86129.47 pmid:16470155
21. Paediatric Association of the Netherlands (NVK). oproep melden casus levenseindezorg 1-12 jaren. 2016 [cited 2019 Oct 08]. Available from: https://www.nvk.nl/Nieuws/articleType/ArticleView/articleId/1707/Oproep-melden-casus-levenseindezorg-1-12-jarigen
22. Brouwer M. Maeckelberghe E, Verhagen E. Beslissingen en Zorg voor Kinderen met een Levenbedreigende Aandoening, Project Website. 2017, [cited 2019 Oct 08]. Available from: https://levensbedreigendziekkind.wordpress.com
23. Sandelowski M. What's in a name? Qualitative description revisited. *Res Nurs Health* 2010;33:77-84
24. Hennink M, Hutter I, Bailey A. Qualitative research methods. Thousand Oaks, CA, Sage; 2010 Dec 8
25. Government of the Netherlands. Wet toetsing levensbeëindiging op verzoek en hulp bij zelfdoding. [Termination of life on request and assisted suicide (Review Procedures) Act]. 2014. [cited 2019 Oct 08]. Available from: http:// wetten.overheid.nl/BWBR0012410/2014-02-15
26. Paediatric Association of the Netherlands (NVK). Richtlijn Palliatieve Zorg voor Kinderen. 2018. [cited 2019 Oct 08]. Available from: http://richtlijnendatabase.nl/richtlijn/palliatieve_zorg_voor_kinderen

For peer review only - http://bmjopen.bmj.com/site/about/guidelines.xhtml

27. Meyer EC, Sellers DE, Browning DM, et al. Difficult conversations: improving communication skills and relational abilities in health care. Pediatr Crit Care Med 2009;10:352-9. doi:10.1097/PCC.0b013e3181a3183a pmid:19325506
28. Hancock K, Clayton J, Parker S, et al. Truth telling in discussing prognosis in advanced life-limiting illnesses: a systematic review. Palliat Med. 2007;21:507-17. doi: 10.1177/0269216307080823 pmid:17846091
29. Crosnier-Schoedel C, Trocmé N, Carbajal R, et al. Pediatrician's experience in announcing bad news. Arch Pediatr. 2018;25:100-6. doi: 10.1016/j.arcped.2017.11.006 pmid:29254736
30. Baile WF, Lenzi R, Kudelka AP, et al. Improving physician—patient communication in cancer care: Outcome of a workshop for oncologists. J Cancer Educ 1997;12:166-73.
31. Coad J, Patel R, Murray S. Disclosing terminal diagnosis to children and their families: Palliative professionals' communication barriers. Death Stud 2014;38:302-7. doi: 10.1080/07481187.2012.753555 pmid:24593008
32. Afonso SB, Minayo MC. Relationships between oncohematopediatrics, mothers and children in communicating bad news. Cien Saude Colet 2017;22:53-62. doi: 10.1590/1413-81232017221.14592016 pmid:28076529
33. Kassam A, Skiadaresis J, Alexander S, et al. Differences in end-of-life communication for children with advanced cancer who were referred to a palliative care team. Pediatr Blood Cancer 2015;62:1409-13. doi: 10.1002/pbc.25530 pmid:25882665
34. Bally JM, Smith NR, Holtslander L, et al. A metasynthesis: Uncovering what is known about the experiences of families with children who have life-limiting and life-threatening illnesses. J Pediatr Nurs 2018;38:88-98. doi: 10.1016/j.pedn.2017.11.004 pmid:29357986
35. Barry MJ, Edgman-Levitan S. Shared decision making—the pinnacle of patient-centered care. N Engl J Med 2012;366:780-1. doi:10.1056/NEJMp1109283 pmid:22375967
36. Zaal-Schuller IH, Willems DL, Ewals FVPM, et al. How parents and physicians experience end-of-life decision-making for children with profound intellectual and multiple disabilities. Res Dev Disabil 2016;59:283-93. doi:10.1016/j.ridd.2016.09.012 pmid:27665411
37. de Vos MA, Bos AP, Plötz FB, et al Talking with parents about end-of-life decisions for their children. Pediatrics. 2015;135(2):e465-76. doi:10.1542/peds.2014-1903 pmid: 25560442
38. Madrigal VN, Carroll KW, Hexem KR, et al. Parental decision-making preferences in the pediatric intensive care unit. Crit care med. 2012;40(10):2876-82. doi:10.1097/CCM.0b013e31825b9151 pmid:22824932
39. Badarau DO, Ruhe K, Kühne T, et al. Decision making in pediatric oncology: Views of parents and physicians in two European countries. AJOB Empir Bioeth. 2017;8:21-31. doi:10.1080/23294515.2016.1234519 pmid:28949870
40. Kane HL, Halpern MT, Squiers LB, et al. Implementing and evaluating shared decision making in oncology practice. CA Cancer J Clin 2014;64:377–88. doi:10.3322/caac.21245 pmid:25200391

For peer review only - http://bmjopen.bmj.com/site/about/guidelines.xhtml

41. Paediatric Association of the Netherlands (NVK). Richtlijn Palliatieve Zorg voor Kinderen [Guidelines on Palliative Care for Children]. 2018 [cited 2019 Oct 08]. Available from: http://richtlijnendatabase.nl/richtlijn/palliatieve_zorg_voor_kinderen

42. Beckstrand RL, Rawle NL, Callister L, et al. Pediatric nurses' perceptions of obstacles and supportive behaviors in end-of-life care. Am J Crit Care 2010;19:543-52. doi:10.4037/ajcc2009497 pmid:20026650

43. Van der Heide A, Onwuteaka-Philipsen BD, Rurup ML, et al. End-of-life practices in the Netherlands under the Euthanasia Act. N Engl J Med 2007;356:1957-65. doi:10.1056/NEJMsa071143 pmid:17494928

44. Verhagen E, Sauer PJ. The Groningen protocol—euthanasia in severely ill newborns. N Engl J Med 2005;352:959-62. doi: 10.1056/NEJMp058026 pmid:15758003

45. Paediatric Association of the Netherlands (NVK). Standpunt NVK Levensbeëindiging kinderen 1-12 jaar: een pleidooi voor onderzoek. 2015. [cited 2019 Oct 08]. Available from: https://www.rijksoverheid.nl/documenten/publicaties/2016/04/22/standpunt-nvk-levensbeeindiging-kinderen-1-12-jaar-een-pleidooi-voor-onderzoek

46. Government of the Netherlands. Kamerbrief met reactie op NVK standpunt levensbeëindiging kinderen. 2016. [cited 2019 Oct 08]. Available from: https://www.rijksoverheid.nl/documenten/kamerstukken/2016/04/22/kamerbrief-met-reactie-op-nvk-standpunt-levensbeeindiging-kinderen

47. Rushton C. Integrating ethics and palliative care in pediatrics. Am J Nurs 2004;104:54–63. doi: 10.1097/00000446-200404000-00019 pmid:15171116

48. Hilhorst M, Deugden van de arts. In: Sanderse W, Kole J, eds. Karakter, Deugden voor professionals. Leusden: ISVW uitgevers 2018:103-12

49. Cheng Y, Lin CP, Liu HL, et al.Expertise modulates the perception of pain in others. Curr Biol. 2007;17:1708-13. doi:10.1016/j.cub.2007.09.020 pmid:17900903

50. Haque OS, Waytz A. Dehumanization in medicine: Causes, solutions, and functions. Perspect Psychol Sci. 2012;7:176-86. doi: 10.1177/1745691611429706 pmid:26168442

51. Haslam N. Humanising medical practice: the role of empathy. Med j Aust. 2007;187:381-2. doi: 10.5694/j.1326-5377.2007.tb01305.x pmid:17907998

For peer review only

Figure 1. Participants' places of residence

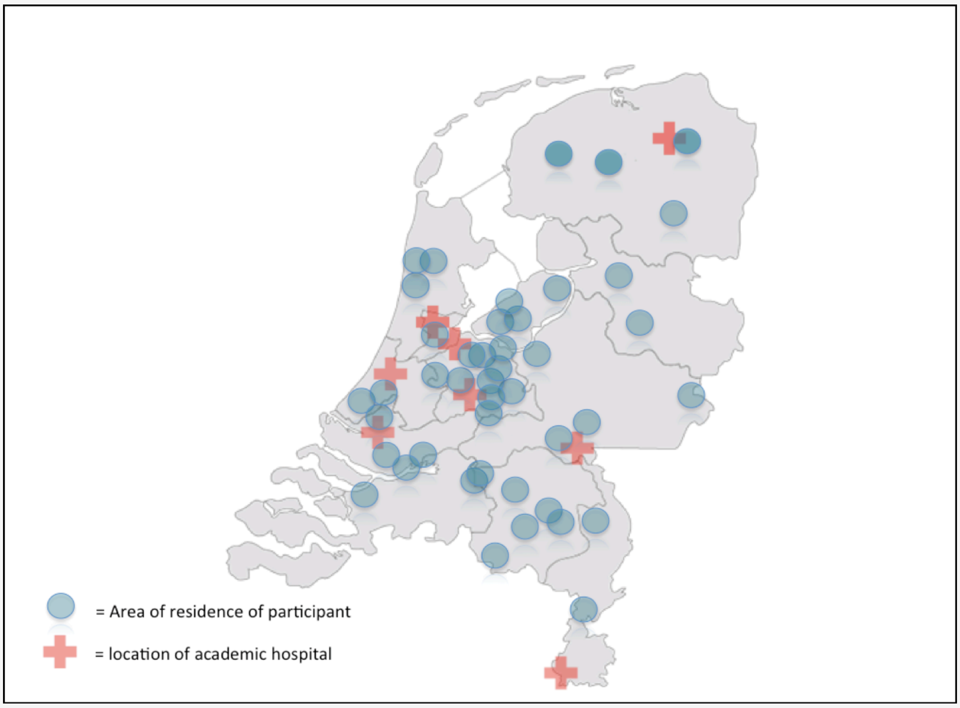

review only

Appendix: Topic guide

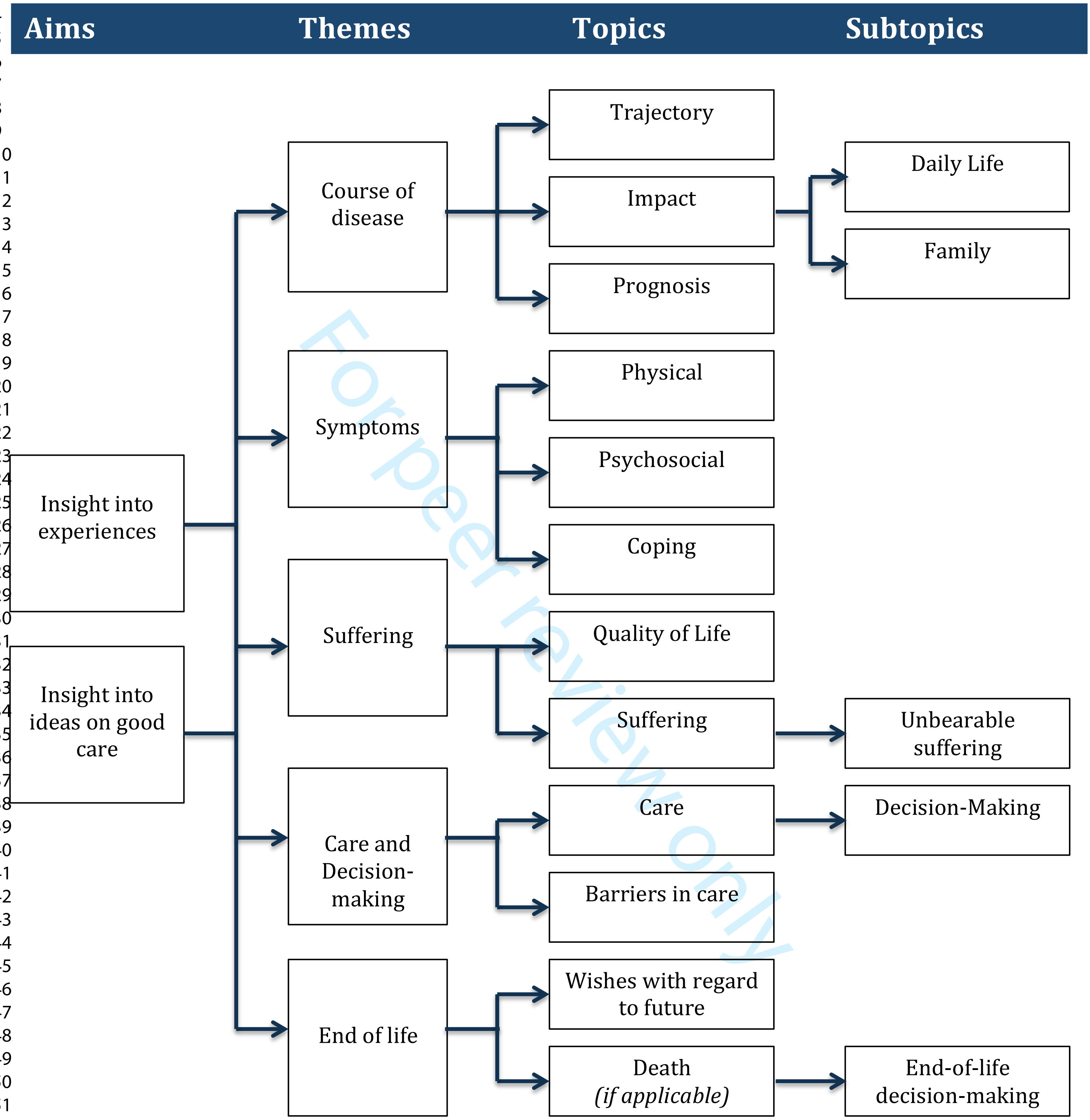

For peer review only

Consolidated criteria for reporting qualitative studies (COREQ): 32-item checklist

Developed from:
Tong A, Sainsbury P, Craig J. Consolidated criteria for reporting qualitative research (COREQ): a 32-item checklist for interviews and focus groups. *International Journal for Quality in Health Care*. 2007. Volume 19, Number 6: pp. 349 – 357

YOU MUST PROVIDE A RESPONSE FOR ALL ITEMS. ENTER N/A IF NOT APPLICABLE

No. Item	Guide questions/description	Reported on Page #
Domain 1: Research team and reflexivity		
Personal Characteristics		
1. Interviewer/facilitator	Which author/s conducted the inter view or focus group?	First author Methods, p. 5
2. Credentials	What were the researcher's credentials? E.g. PhD, MD	MA Methods, p.5
3. Occupation	What was their occupation at the time of the study?	PhD student Methods, p.5
4. Gender	Was the researcher male or female?	Female Methods, p.5
5. Experience and training	What experience or training did the researcher have?	training Methods, p.5
Relationship with participants		
6. Relationship established	Was a relationship established prior to study commencement?	none Methods, p.5
7. Participant knowledge of the interviewer	What did the participants know about the researcher? e.g. personal goals, reasons for doing the research	Nothing Methods, p.5
8. Interviewer characteristics	What characteristics were reported about the inter viewer/facilitator? e.g. Bias, assumptions, reasons and interests in the research topic	None Methods, p.5
Domain 2: study design		
Theoretical framework		
9. Methodological orientation and Theory	What methodological orientation was stated to underpin the study? e.g. grounded theory, discourse analysis, ethnography, phenomenology, content analysis	Thematic analysis using constant comparative method. Methods, p.6
Participant selection		
10. Sampling	How were participants selected? e.g. purposive, convenience, consecutive, snowball	Maximum variety Methods, p.5
11. Method of approach	How were participants approached? e.g. face-to-face, telephone, mail, email	Various. See Methods, p.5

For peer review only

12. Sample size	How many participants were in the study?	64 parents of 44 children.<br><br>Results, see p.6
13. Non-participation	How many people refused to participate or dropped out? Reasons?	3, Methods. p.5
Setting		
14. Setting of data collection	Where was the data collected? e.g. home, clinic, workplace	Methods, interviews. p. 5
15. Presence of non-participants	Was anyone else present besides the participants and researchers?	Some parents were interviewed together with their spouse.<br>p.5
16. Description of sample	What are the important characteristics of the sample? e.g. demographic data, date	See Results, table 1 and 2. (p.6, p.7)
Data collection		
17. Interview guide	Were questions, prompts, guides provided by the authors? Was it pilot tested?	Methods, interviews see p.2
18. Repeat interviews	Were repeat inter views carried out? If yes, how many?	No.<br>Methods, p.5
19. Audio/visual recording	Did the research use audio or visual recording to collect the data?	Audio records<br><br>Methods, p.5
20. Field notes	Were field notes made during and/or after the inter view or focus group?	N/A
21. Duration	What was the duration of the inter views or focus group?	On average 2 hours.<br><br>Methods, p.5
22. Data saturation	Was data saturation discussed?	Yes<br>Methods, p.5
23. Transcripts returned	Were transcripts returned to participants for comment and/or correction?	Yes,<br>Methods. p.5
Domain 3: analysis and findings		
Data analysis		
24. Number of data coders	How many data coders coded the data?	Two main coders, checked by all 5 authors.<br>Methods see p.5
25. Description of the coding tree	Did authors provide a description of the coding tree?	See results, p.6. The coding tree can be added to the manuscript if needed.
26. Derivation of themes	Were themes identified in advance or derived from the data?	From the data.<br>See methods. p.5
27. Software	What software, if applicable, was used to	Atlas.ti,

For peer review only

	manage the data?	Methods, data analysis, see p.5
28. Participant checking	Did participants provide feedback on the findings?	By means of an advisory board.<br><br>Methods, p.6
Reporting		
29. Quotations presented	Were participant quotations presented to illustrate the themes/findings? Was each quotation identified? e.g. participant number	Yes, See results.
30. Data and findings consistent	Was there consistency between the data presented and the findings?	See results and discussion
31. Clarity of major themes	Were major themes clearly presented in the findings?	See Results
32. Clarity of minor themes	Is there a description of diverse cases or discussion of minor themes?	See results.

For peer review only

Standards for Reporting Qualitative Research (SRQR)

O'Brien B.C., Harris, I.B., Beckman, T.J., Reed, D.A., & Cook, D.A. (2014). Standards for reporting qualitative research: a synthesis of recommendations. *Academic Medicine, 89(9)*, 1245-1251.

No. Topic	Item
**Title and abstract**	
S1 Title<br><br>Concise description of the nature and topic of the study identifying the study as qualitative or indicating the approach (e.g., ethnography, grounded theory) or data collection methods (e.g., interview, focus group) is recommended	The Title of the manuscript is:<br><br>*Barriers in Care for Children With Life-Threatening Conditions: a Qualitative Interview Study in he Netherlands*
S2 Abstract<br><br>Summary of key elements of the study using the abstract format of the intended publication; typically includes objective, methods, results, and conclusions	The key elements of the study (objective, design, setting, participants results and conclusions) have been summarized in the abstract. (see p.2)
**Introduction**	
S3 Problem formulation<br><br>Description and significance of the problem/phenomenon studied; review of relevant theory and empirical work; problem statement	The main problem is that in current literature on pediatric palliative care, knowledge of parents' experiences of barriers to good pediatric palliative care is limited. This is stated in the introduction (see p.2)
S4 Purpose or research question<br><br>Purpose of the study and specific objectives or questions	The purpose of the study is to provide insight into barriers to care and decision-making for children living with life-threatening conditions. (see p.2)
**Methods**	
S5 Qualitative approach and research paradigm<br><br>Qualitative approach (e.g., ethnography, grounded theory, case study, phenomenology, narrative research) and guiding theory if	The qualitative approach used in this study is qualitative description, and a thematic analysis was performed using the constant comparative approach. (see p.6)

For peer review only

appropriate; identifying the research paradigm (e.g., positivist, constructivist/interpretivist) is also recommended	
S6 Researcher characteristics and reflexivity<br><br>Researchers' characteristics that may influence the research, including personal attributes, qualifications/experience, relationship with participants, assumptions, or presuppositions; potential or actual interaction between researchers' characteristics and the research questions, approach, methods, results, or transferability	Characteristics of the interviewer are described in methods section interviews (see p.5)<br>Credentials:MA<br>Position: PhD student<br>Sex: female<br>education in interviewing: formal training<br><br>The analysis team consisted of a PhD student, an ethicist, a professor in end-of-life care, and a child psychiatrist and a physician/professor in paediatric palliative care. This is described in the methods section (see p.6)
S7 Context<br><br>Setting/site and salient contextual factors; rationale[a]	Participants were usually interviewed at their place of residence. They were free in conducting the interview alone or together (see p.5)
S8 Sampling strategy<br><br>How and why research participants, documents, or events were selected; criteria for deciding when no further sampling was necessary (e.g., sampling saturation); rationale[a]	. Sampling was aimed at obtaining maximum variety in terms of health condition, age, cultural background, parental level of education and place of residence. (see p.5)<br><br>To recruit participants, all Dutch paediatricians received an invitation from the Paediatric Association of the Netherlands (NVK) to invite potential parents to join the research. A website with information about the study was created to inform potential participants. Parent support groups in oncology, cardiology, metabolic and neuromuscular diseases and palliative care helped to reach potential participants through their online platforms. To ensure maximum variety in ethnic background we recruited through physicians with expertise in treating patients with different cultural backgrounds. (see p.5)<br><br>Sampling continued until thematic saturation and saturation of variety in participants was achieved. (see p.5)
S9 Ethical issues pertaining to human subjects<br><br>Documentation of approval by an appropriate ethics review board and participant consent, or explanation for lack thereof; other confidentiality and data security issues	The study was reviewed and deemed exempt by the medical ethical committee of the University Medical Center Groningen. (see p.5)
S10 Data collection methods<br><br>Types of data collected; details of data collection procedures including (as appropriate) start and stop dates of	Parents were interviewed in face-to-face in-depth interviews.

For peer review only

data collection and analysis, iterative process, triangulation of sources/methods, and modification of procedures in response to evolving study findings; rationale[a]	
S11 Data collection instruments and technologies<br><br>Description of instruments (e.g., interview guides, questionnaires) and devices (e.g., audio recorders) used for data collection; if/how the instrument(s) changed over the course of the study	A topic guide had been developed as an interview guide. The interview covered the following themes: course of the disease, aspects of the illness (symptoms and impact) suffering, care, decision-making and end of life. The topic guide is added as a supplementary file to the manuscript. (see p.5)
S12 Units of study<br><br>Number and relevant characteristics of participants, documents, or events included in the study; level of participation (could be reported in results)	In the results section, the relevant characteristics of the participants are described: characteristics of children: status, age, gender, diagnosis<br>Characteristics of parents: relationship status, education, ethnicity, spiritual beliefs, family composition.<br><br>(see p.7 table 1 and 2)
S13 Data processing<br><br>Methods for processing data prior to and during analysis, including transcription, data entry, data management and security, verification of data integrity, data coding, and anonymization/deidentification of excerpts	The interviews were recorded on audio and subsequently transcribed verbatim. All interviews were anonymized during transcription. Participants were given the opportunity to revise their transcribed interviews. (see. p.5)
S14 Data analysis<br><br>Process by which inferences, themes, etc., were identified and developed, including researchers involved in data analysis; usually references a specific paradigm or approach; rationale[a]	A primary thematic analysis, aiming at a qualitative description of barriers as perceived by parents, was performed using the constant comparative approach.
S15 Techniques to enhance trustworthiness<br><br>Techniques to enhance trustworthiness and credibility of data analysis (e.g., member checking, audit trail, triangulation); rationale[a]	Coding was performed by the first two authors and reviewed by all authors. Differences were settled by discussion until consensus was achieved. During the study, biannual meetings were held with an advisory board (9 members) of parents, physicians and researchers who provided feedback on the findings. (see. p.6)
**Results/Findings**	
S16 Synthesis and interpretation<br><br>Main findings (e.g., interpretations, inferences, and themes); might include development of a theory or model, or integration with prior research or theory	As the theoretical framework of this study is qualitative decription, we did not include a theoretical model in this transcript. The results section discusses the main barriers as experienced by parents (see results section)
S17 Links to empirical data	In the results section, quotes are used to illustrate the findings. (see

For peer review only

Evidence (e.g., quotes, field notes, text excerpts, photographs) to substantiate analytic findings	results section)
**Discussion**	
S18 Integration with prior work, implications, transferability, and contribution(s) to the field<br><br>Short summary of main findings; explanation of how findings and conclusions connect to, support, elaborate on, or challenge conclusions of earlier scholarship; discussion of scope of application/generalizability; identification of unique contribution(s) to scholarship in a discipline or field	In the discussion, the main findings are discussed (see p.14) and placed in the context of exiting literature. (see p. 14- 17)
S19 Limitations<br><br>Trustworthiness and limitations of findings	The discussion contains a limitations section discussing relevant limitations of this study. (see p. 17)
**Other**	
S20 Conflicts of interest<br><br>Potential sources of influence or perceived influence on study conduct and conclusions; how these were managed	Disclosure of interests. The authors have no conflict of interest or financial relations to disclose. (see p. 18)
S21 Funding<br>Sources of funding and other support; role of funders in data collection, interpretation, and reporting	This work was supported by the Dutch Ministry of Health, Welfare and Sport. Grant number 324717 (560019968)] (see p.18)

[a]The rationale should briefly discuss the justification for choosing that theory, approach, method, or technique rather than other options available, the assumptions and limitations implicit in those choices, and how those choices influence study conclusions and transferability. As appropriate, the rationale for several items might be discussed together.

For peer review only

BMJ Open

Barriers in Care for Children With Life-Threatening Conditions: a Qualitative Interview Study in the Netherlands

Journal:	*BMJ Open*
Manuscript ID	bmjopen-2019-035863.R2
Article Type:	Original research
Date Submitted by the Author:	05-May-2020

Complete List of Authors:	Brouwer, Marije; Universitair Medisch Centrum Groningen, pediatrics<br>Maeckelberghe, Els; Universitair Medisch Centrum Groningen, Institute for Medical Education FC 40<br>van der Heide, Agnes; Erasmus MC, University Medical Center Rotterdam, Clinical Microbiology<br>Hein, Irma; Academic Medical Center: Amsterdam, psychiatry<br>Verhagen, Eduard; University Medical Center Groningen, University of Groningen, Dept of Pediatrics

**Primary Subject Heading**:	Paediatrics
Secondary Subject Heading:	Palliative care, Ethics
Keywords:	PALLIATIVE CARE, MEDICAL ETHICS, Paediatric palliative care < PALLIATIVE CARE

SCHOLARONE™
Manuscripts

I, the Submitting Author has the right to grant and does grant on behalf of all authors of the Work (as defined in the below author licence), an exclusive licence and/or a non-exclusive licence for contributions from authors who are: i) UK Crown employees; ii) where BMJ has agreed a CC-BY licence shall apply, and/or iii) in accordance with the terms applicable for US Federal Government officers or employees acting as part of their official duties; on a worldwide, perpetual, irrevocable, royalty-free basis to BMJ Publishing Group Ltd ("BMJ") its licensees and where the relevant Journal is co-owned by BMJ to the co-owners of the Journal, to publish the Work in this journal and any other BMJ products and to exploit all rights, as set out in our licence.

The Submitting Author accepts and understands that any supply made under these terms is made by BMJ to the Submitting Author unless you are acting as an employee on behalf of your employer or a postgraduate student of an affiliated institution which is paying any applicable article publishing charge ("APC") for Open Access articles. Where the Submitting Author wishes to make the Work available on an Open Access basis (and intends to pay the relevant APC), the terms of reuse of such Open Access shall be governed by a Creative Commons licence – details of these licences and which Creative Commons licence will apply to this Work are set out in our licence referred to above.

Other than as permitted in any relevant BMJ Author's Self Archiving Policies, I confirm this Work has not been accepted for publication elsewhere, is not being considered for publication elsewhere and does not duplicate material already published. I confirm all authors consent to publication of this Work and authorise the granting of this licence.

For peer review only

Barriers in Care for Children With Life-Threatening Conditions: a Qualitative Interview Study in the Netherlands

MARIJE BROUWER, ELS MAECKELBERGHE, AGNES VAN DER HEIDE, IRMA HEIN, and EDUARD VERHAGEN

Marije A. Brouwer, MA
m.a.brouwer@umcg.nl
Department of paediatrics, University of Groningen, University Medical Center Groningen, Groningen, the Netherlands

dr. Els L.M. Maeckelberghe
e.l.m.maeckelberghe@umcg.nl
Institute for Medical Education, University of Groningen, University Medical Center Groningen, Groningen, the Netherlands

prof. Agnes van der Heide
a.vanderheide@erasmucmc.nl
Department of Public Health, Erasmus medical Center, Rotterdam, the Netherlands

dr. Irma M. Hein
i.hein@debascule.com
Department of psychiatry, Academic Medical Center, Amsterdam, the Netherlands

prof. A.A.E. Eduard Verhagen
a.a.e.verhagen@umcg.nl
Department of paediatrics, University of Groningen, University Medical Center Groningen, Groningen, the Netherlands

Address correspondence to:
Marije Aafke Brouwer
University Medical Center Groningen, Department of Pediatrics,
tnv A.J.Heinstra/M.A.Brouwer.
Antwoordnummer 333, 9700 VB Groningen
mail: m.a.brouwer@umcg.nl
phone: 0031-621102049

Word count (excluding abstract, references, and quotes): 4015

For peer review only

Abstract.

Objective: To identify barriers, as perceived by parents, to good care for children with life-threatening conditions.

Design: In a nationwide qualitative study, we held in-depth interviews regarding end-of-life care with parents of children (aged one to 12 years) who were living with a life-threatening illness or who had died after a medical trajectory (a maximum of five years after the death of the child). Sampling was aimed at obtaining maximum variety for a number of factors. The interviews were transcribed and analysed.

Setting: The Netherlands.

Participants: 64 parents of 44 children.

Results: Parents identified six categories of difficulties that create barriers in care for children with a life-threatening condition. First, parents wished for more empathetic and open communication about the illness and prognosis. Second, organizational barriers create bureaucratic obstacles and a lack of continuity of care. Third, parents wished for more involvement in decision-making. Fourth, parents wished they had more support from the health care team on end-of-life decision-making. Fifth, parents experienced a lack of attention for the family during the illness and after the death of their child. Sixth, parents experienced an overemphasis on symptom-treatment and lack of attention for their child as a person.

Conclusions: The barriers as perceived by parents focused almost without exception on non-medical aspects: patient-doctor relationships, communication, decision-making, including end-of-life decision-making, and organization. The perceived barriers indicate that care for children with a life-threatening condition focuses too much on symptoms and not enough on the human beings behind these symptoms.

Strengths and limitations.

Strengths:

- The variety: Parents from children with various illnesses, ages and prognosis, receiving care from various hospitals were included.
- The size: 64 participating parents from 44 children were interviewed, and over 110 hours of qualitative data on audio were collected.

Limitations:

- The study focuses on young children (aged one to 12 years old) and the results cannot be generalized to adolescents or neonates.

For peer review only - http://bmjopen.bmj.com/site/about/guidelines.xhtml

- The success in achieving variation in the cultural and religious background of participants with different cultural and religious backgrounds variation was limited.
- The study reports only the barriers experiences by parents.

For peer review only

For peer review only - http://bmjopen.bmj.com/site/about/guidelines.xhtml

Introduction.

Parents and physicians caring for children with life-threatening conditions face multiple challenges in providing the best care for children. Care for children whose futures and chances of survival are uncertain is often a complex trajectory, where curative and palliative care entwines.[1, 2] Curative care goals – to cure/prolong life – and palliative care goals – to relieve suffering – sometimes collide, complicating care. Care for these children is further complicated because some decisions to reduce the child's suffering in the context of palliative may also limit the life-expectancy of a child (such as discontinuing or reducing life-sustaining technology).[3, 4]

Life-threatening conditions are defined as conditions for which curative treatment may be feasible, but for which this treatment could also fail, leading to a possibility of dying.[5] Care for children with life-threatening conditions can include aspects of both curative and palliative care at the same time.[2] Healthcare providers are not always confident about their skills in providing palliative care.[6] The main reported barriers in paediatric palliative care as experienced by healthcare providers are communication, the uncertainty of the prognosis, time constraints and lack of education.[7-11] Studies on barriers experienced by parents show that communication is a major barrier for them.[12-17] Other frequently mentioned barriers are a lack of care for the family[12-13] and bereavement care.[17]

But knowledge of parents' experiences of barriers to good care is limited in at least two ways. First, it focuses mainly on specific health conditions, particularly on oncology,[14,15] and second, it focuses mainly on children receiving palliative care, often with a certain, lethal prognosis.[12-13,16-17] However, not all children who have life-threatening conditions receive palliative care, or are diagnosed with a lethal prognosis.[18-20]

To provide insight into barriers to care and decision-making for children living with life-threatening conditions, we conducted a qualitative study throughout the Netherlands. We interviewed parents who had experience in caring for a child with a life-threatening condition to identify what they regarded as barriers to both good care and good decision-making.

Methods.

For peer review only - http://bmjopen.bmj.com/site/about/guidelines.xhtml

In a large-scale, nationwide qualitative study we interviewed parents of children, either living with a life-threatening or terminal condition, or who had died after a medical trajectory (a maximum of five years before the date of the interview). Children were aged between one and 12 years old and had a variety of life-threatening conditions. The study was reviewed and deemed exempt by the medical ethical committee of the University Medical Center Groningen.

Recruitment.

Participants were recruited between November 2016 and October 2018. To recruit participants, all Dutch paediatricians received an invitation from the Paediatric Association of the Netherlands (NVK) to invite potential parents to join the research.[21] A website with information about the study was created to inform potential participants.[22] Parent support groups in oncology, cardiology, metabolic and neuromuscular diseases and palliative care helped to reach potential participants through their online platforms. To ensure maximum variety in ethnic background we recruited through physicians with expertise in treating patients with different cultural backgrounds. Sampling was aimed at obtaining maximum variety in terms of health condition, age, cultural background, parental level of education and place of residence. Three participants dropped out due to scheduling difficulties. Recruitment continued until thematic saturation and saturation of variety in participants was achieved.

Interviews.

Parents were interviewed in face-to-face in-depth interviews, usually held at the parent's place of residence. The first author (MA, PhD student, female) conducted the interviews after following formal training. The participants signed an informed consent form before the interview. Parents did not know the interviewer before the study. All participants were given the choice to conduct the interview alone or together with a co-parent. A topic guide had been developed as an interview guide. The interview covered the following themes: course of the disease, symptoms, suffering, care and decision-making and end of life. The topic guide is added as a supplementary file to the manuscript. The interviews were recorded on audio and subsequently transcribed verbatim. All interviews were anonymized during transcription. The average duration of the interviews was two hours. Participants were given the opportunity to revise their transcribed interviews.

Analysis.

For peer review only - http://bmjopen.bmj.com/site/about/guidelines.xhtml

For this article, we selected quotes concerning perceived barriers in care. For reasons of practicality in handling the approximately 2,500 pages of transcripts, the first author gathered all codes on barriers in care in parent interviews, which were subsequently read by all authors to become familiarized with the content. The first author further focused on parents' perceptions of barriers in care in the selection of interview fragments, and this was reviewed by all authors. The research team (the authors of this paper) consisted of a PhD student, an ethicist, a professor in end-of-life care, and a child psychiatrist and a physician/professor in paediatric palliative care.

A primary thematic analysis, aiming at a qualitative description of barriers as perceived by parents,[23] was performed using the constant comparative approach.[24] Coding was performed by the first two authors and reviewed by all authors. Differences were settled by discussion until consensus was achieved.

Patient and Public Involvement.

The focus of this study is on parent experiences and preferences. During the study, biannual meetings were held with an advisory board (9 members) of parents, physicians and researchers who provided feedback on the findings. They were offered remuneration for their efforts. They provided substantial input on the study design, recruitment, analysis and the reporting of results of the study. All participants were given the opportunity to check and revise their manuscript, and are regularly updated on the outcomes of the study by e-mail.

Results.

Participants. We interviewed 64 parents of 44 children. Our participants came from all over the Netherlands. All children received, or had received, care in at least one university medical centre, often combined with care in one or more local hospitals. All eight university hospitals in the Netherlands, as well as over 20 local hospitals, were represented in the study. Characteristics of children and parents are shown in Tables 1,2 and in Figure 1.

Table 1. Characteristics of children (n=44)

Status, deceased	27 (61.4%)
Gender, male	20 (45.5%)
Age (years)	

For peer review only - http://bmjopen.bmj.com/site/about/guidelines.xhtml

1-3	15 (34.0%)
4-6	8 (18.2%)
7-9	9 (20.5%)
10-12	12 (27.3%)
Diagnosis	
Malignancies	18 (40.9%)
Neurological/metabolic	17 (38.6%)
Cardiovascular	4 (9.1%)
CNS	3 (6.8%)
Other	1 (2.2%)
Undiagnosed	1 (2.2%)

Table 2. Characteristics of parents (n=64 interviewed parents, 42 couples

Relationship status	
Married/Together	34 (81.0%)
Level of education, mothers (N=42)[1]	
Low-level education (< 4 years)	1 (2.4%)
Mid-level education (practical, 4 years)	15 (35.7%)
Higher education (vocational, 4 years),	14 (33.3%)
Academic education (University, 4-6 years)	12 (28.6%)
Level of education, fathers (N=42)[1]	
Low-level education (< 4 years)	4 (9.5%)
Mid-level education (practical, 4 years)	15 (35.7%)
Higher education (vocational, 4 years),	14 (33.3%)
Academic education (University, 4-6 years)	9 (21.4%)
Ethnicity of parents, according to the participants (N=42)	
Dutch	36 (85.7%)
Mixed	6 (14.3%)
Religious/ spiritual beliefs (N=64)	
None	39 (60.9%)
Christian	19 (29.7%)
Other	6 (9.4%)
Family composition	
1 child	8 (19.0%)
2 children	22 (52.4%)
3 children	10 (23.8%)
4 or more children	2 (4.8%)

[1.] We considered the educational level of both parents to be possibly of influence on the care and decisions of the child. Therefore the educational level of both parents is represented, even if one of them was not interviewed.

<figure 1>

For peer review only - http://bmjopen.bmj.com/site/about/guidelines.xhtml

Experienced barriers. We identified six major barriers in care for children with life-threatening conditions: We summarized the barriers in Table 3.

Table 3. Barriers in paediatric palliative care, as experienced by parents.

**Barrier 1. Parents wished for more empathic and open communication about the illness and prognosis.**
1a. Absence of conversations about the future and possible prognosis.
1b. Communication lacks empathy.
**Barrier 2: Organizational barriers create bureaucratic obstacles and a lack of continuity of care.**
2a. Lack of continuity in care threatens good care
2b. Healthcare structures create bureaucratic obstacles.
**Barrier 3. Parents wished for more involvement in decision-making**
**Barrier 4. Parents wished they had more support from the health care team on end-of-life decision making**
**Barrier 5. Parents experienced a lack of attention for the family during the illness and after the death of their child.**
5a. Impact on parents and siblings is neglected in care.
5b. Parents experience a lack of bereavement care.
**Barrier 6. Parents experience an overemphasis on symptom-treatment and lack of attention for their child as a person.**
6a. Lack of attention for how a child might experience treatment and illness.
6b. Failure to evaluate symptoms in the context of the child's illness as a whole.
6c. Overemphasizing the patients role as a sick person instead of a human being .

Barrier 1: Parents wished for more empathetic and open communication about the illness and prognosis

Parents univocally felt that the wish to receive complete and open information, and the absence thereof, was a significant barrier to good care. Most experiences regarding communication involved either a perceived lack of openness about the prognosis or a lack of empathy in communication.

1a. Absence of conversations about the future and possible prognosis.

Telling a parent that their child may not survive might seem like a message that parents would not like to receive, but parents (both bereaved parents and non-bereaved parents) stressed that they had wished for open conversations about their child's future. Not talking about the

For peer review only - http://bmjopen.bmj.com/site/about/guidelines.xhtml

prognosis creates uncertainty and makes the topic a taboo. They underlined the importance to openly talk about this, especially when the prognosis is uncertain.

> (F02): 'In the first few years, you still have the hope that things might change, but after a few years, you realize that almost all of that hope has gone. It would be great if physicians would take you by the hand and say: "We have to accept that this is what it is, and that things are probably not going to get better." And ask you as a parent: "What are your thoughts on that? Would you, despite that prognosis, like to keep on trying new treatments? Or do you think it is better not to do that?"'

Parents specifically needed openness about the prognosis to discuss the possibility of transition between different levels of concurrent life-prolonging treatments and comfort care, or to make end-of-life decisions. In a few cases, the topic of dying was only mentioned a day before the death of their child.

1b. Communication lacks empathy. Parents experienced a lack of empathetic communication. They felt that the friction between healthcare professionals doing their daily jobs and families going through a once-in-a-lifetime event was characteristic for the lack of sympathy that they experienced.

> (M27): '[the lady who came to explain the procedure of palliative sedation] was so insensitive. She just explained all the technicalities, with a broad smile. And I remember thinking: "It's not like I am buying bread from you!" (…) It was as if she thought: "let's get this over with", as if there was not an entire life, an entire family, an entire world affected by the procedure that she was explaining.'

But experienced barriers in communication were not limited to interactions with healthcare providers or hospital settings.

> (F16): 'Before we had even held the funeral, we received a letter from the local authority asking us to return our special parking permits as soon as possible. Just like that, without any condolences. Bizarre.'

Barrier 2: Organizational barriers create bureaucratic obstacles and a lack of continuity of care.

With regard to organization, two themes emerged: continuity of care and bureaucracy.

For peer review only - http://bmjopen.bmj.com/site/about/guidelines.xhtml

2a. Lack of continuity in care threatens quality. Parents described having to deal with many different care providers, either individual professionals or organizations, which made it difficult to guarantee continuity of care. Agreements made with one provider did not always dovetail well with the care that they received from other care providers.

> (F03): 'There was a physician on duty, and when we updated him on the 'do not resuscitate' agreements that we had made with our regular physician, he said: "No, I'm not going to do that. It is my shift, and my responsibility, and I'm not going to do that." You take all this time to make decisions with your doctor, and then another doctor, who happens to be on duty and doesn't know your child or the file, who has never had a conversation with you, simply states: "I'm responsible, so I determine what happens."'

2b. Healthcare structures create bureaucratic obstacles.. Parents particularly experienced bureaucratic barriers to good care in situations where they had to deal with a multitude of healthcare-related organizations.

> (M31) 'When the neurologist prescribes some sort of medicine, he has to send it by fax to the pharmacist, he can't send it by email. And if there is even one comma in the wrong place, the pharmacist won't prescribe the medicine that my son needs.'

Several parents expressed the need for a case manager, who could bridge the gaps between parents, different healthcare providers and financers. Notably, this barrier was also mentioned by parents who had received palliative care from teams that already worked with case managers. While more and more paediatric palliative teams in the Netherlands are appointing case managers to families dealing with a life-threatening illness, the question arises as to how they can be used in the most effective manner.

Barrier 3: Parents wished for more involvement in decision-making

The third barrier concerned participation in decision-making. There was a lot of variation regarding the involvement of parents in decision-making. Parents who had been involved in decision-making, appreciated it.

> (M15): '[when we had to decide whether to go for radiotherapy or not] our doctor said: "Whatever your decision is, there is no wrong answer, and I will stand by your choice."'

For peer review only - http://bmjopen.bmj.com/site/about/guidelines.xhtml

Other parents described that they were not as involved in decision-making as they would have liked, both for decisions made during curative treatment and for those made in the terminal phase. One parent described that the decision to withdraw life-sustaining treatment for their child was made by a neurologist who had never spoken to them.

> (M27): 'The neurologist never spoke to us about his motivations to [withdraw treatment] and start the death of our son. He came in, made the observation right in front of us, but only spoke in private to our paediatrician, and left.'

All participants stressed the importance of involving parents in the decision-making process, stating that the decisions included considerations about the suffering, quality of life and daily life of the child, something in which the parents' expertise was needed. The only exception parents mentioned to being involved, were decisions that parents saw as 'merely medical' information, such as technicalities about medication or treatment.

Barrier 4. Parents wished they had more support from the health care team at end-of-life decision-making

Parents mentioned discussions about the possibility of transition between different levels of concurrent life-prolonging treatments and comfort care, or to make end-of-life decisions, as major themes in the care for their child. Parents sometimes had doubts about the appropriateness of continued treatment or felt that it was wrong that parents themselves had to initiate a conversation about end-of-life decision-making.

Additionally, several parents expressed a wish for more legal possibilities for active life-ending in order to relieve their child's suffering and grant their child a dignified end of life. In the Netherlands, active life-ending for children – sometimes referred to as paediatric euthanasia – is not legal for children aged between one and 12 years old.[25] Withholding or withdrawing treatment and palliative sedation are considered permitted medical practices.[26]

For the majority of the children in the study, end-of-life decisions were made, ranging from withholding or withdrawing life-prolonging or life-sustaining treatment to palliative sedation. Nevertheless, in several cases, parents still felt the need for an option to make end-of-life decisions. In most cases, their need came from a situation in which the condition of their child

For peer review only - http://bmjopen.bmj.com/site/about/guidelines.xhtml

was physiologically stable and the physicians could not justify the withdrawal of treatment or palliative sedation.

> (M02) 'Physicians told us that if they had to [give her] so much morphine to ease her pain that the morphine itself would be the cause of her death, it would be considered active life-ending, which is not allowed for children who cannot ask for it themselves. But we would want that for her, because we have the feeling that the only thing that can bring her comfort and peace (emotional) will be death.'

In other situations, parents felt a need for active life-ending because the existing options were unable to grant their child a dignified end-of-life.

> (M07): 'If we all know that the palliative sedation is going to result in his death, why does he have to go through these final days of suffering? Why does it have to be so... undignified?'

Barrier 5. Parents experienced a lack of attention for the family during the illness and after the death of their child.

A life-threatening illness does not only affect children, but also their families. Parents felt that they and their families were not always supported during and after the illness of their child. Siblings in particular were often overlooked.

> (M20): 'As a parent you get a little support, but for brothers and sisters there is very little to absolutely no help at all. They have to figure it out for themselves, it seems as though they are forgotten.'

While support for the family is one of the aims of paediatric palliative care,[26] and all Dutch hospitals have psychologists, social workers and/or palliative care teams to provide this, it seems that this support is not always offered to parents or did not function as well as parents had hoped. However, for parents, the need was not so much about offering psychological support as it was about empathy in their contact with the healthcare providers that they came across.

> (M05) 'What they don't always realize is that the hospital also becomes your social world; the contact with doctors, the chats with nurses.'

For peer review only - http://bmjopen.bmj.com/site/about/guidelines.xhtml

Barrier 6. Parents experience an overemphasis on symptom-treatment and lack of attention for their child as a person.

The final barrier expressed by parents was a wish for healthcare providers to understand the fact that patients are persons, not merely carriers of certain symptoms. They explained that healthcare professionals often failed to evaluate the impact of the condition or the medical interventions on the child's daily life. This barrier had three different aspects: 1) a lack of attention for how a child might understand and cope with treatment and illness, 2) failure to see the complexity of the child as a whole and, finally, 3) overemphasizing the child as a patient instead of as a whole person.

6a. Lack of attention for how a child might experience treatment and illness.

One specific characteristic of paediatric care is the attention given to how children understand and cope with their condition and the associated treatment. Parents underlined that this is important both for 'bigger questions' and for simple medical interventions. Parents mentioned the comprehension of the child as a vital aspect in the child's ability to cope with illness and treatment.

> (M04) 'Hospitals are busy with production. So, a nurse comes in, and basically jumps on the child to take his blood pressure, or give an injection, without paying attention to what it does to him (…) And I often wonder: How hard can it be to ask yourself beforehand how to approach this individual child, so that he understands the interventions?'

Parents saw taking the time to explain to children what is happening them (instead of performing treatment on them without explanation) as an important aspect of taking children seriously as people.

6b Failure to evaluate symptoms in the context of the child's illness as a whole.

Children with life-threatening conditions can receive treatment from various specialists. Parents described how specialists sometimes failed to look beyond the specific symptoms that they were treating and see the child and the complexity of their condition as a whole.

> (M29): 'We had an incident because my son had fallen, and the dental surgeon took one look at his teeth and said: "Those teeth have got to go." And I said: "But what then? He already has

For peer review only - http://bmjopen.bmj.com/site/about/guidelines.xhtml

problems swallowing." And he said casually: "He'll need a feeding tube." Not caring what it would mean to him, and our lives, to make that decision. And I don't think he even saw the boy who was sitting there, who I love so much, and who I am so worried about.'

6c. Overemphasizing the patients role as a sick person instead of a human being.

The final barrier identified was the overemphasis of the child as a patient, at the cost of the child as a person. Parents described how medical treatment sometimes overemphasized the child as a patient and that there was little attention for the child as a person, impeding the child's quality of life. Parents stressed that especially for ill children keeping up a normal life became very important: going to school, not standing out, feeling like an ordinary child. Daily activities that did not focus on the child's illness, but on the child as an ordinary person, became cherished. As this mother remembers:

(M14): 'It was the highlight of his day, when his teacher would come; solving math problems (laughs).'

This barrier also influenced the readiness of a child to communicate about their illness. Children did not always want to talk about the illness, a few even forbidding their parents to talk about it with others. Seeing the child too much as a patient, either by focussing merely on the child's illness, or by trying to make them feel special, might deprive children of living a normal life.

Both barriers five and six refer to what parents described as their wish for a more human-centred approach to care, which also implied an empathic connection between professionals and parents/patients. According to the parents, some physicians perceived this attitude as unprofessional:

(M20): 'One physician had a big impact on me. We had to go home, and she said goodbye to my son. She started crying and apologized: "I'm so sorry, I'm being very unprofessional."'

For parents, however, this kind of connection was much appreciated and in fact seen as a sign of professionalism.

For peer review only

(M20, continued): 'And I said: "No, right now, you are human." I wonder if she knows that of all those doctors, she is the only one that crosses my mind frequently. Because she cared.'

Discussion.

Main findings. This qualitative study gives insight into parents' perceived barriers in care for children with life-threatening conditions. The barriers experienced by parents focused almost without exception on the non-technical skills in medicine: attention for children, empathy, communication, decision-making and organization. The absence of perceived barriers on the execution of symptomatic care is notable.

With respect to the first barrier, communication, other studies report communication as a barrier for both physicians [7-8,11,27-31] and parents.[12-16,32-34] Our results underline the importance of openness as a requirement for decision-making, and adds that communication is not limited to hospital settings. A possible future development for paediatric palliative care is to extend communication training to all professionals dealing with children with life-threatening conditions, not merely hospital workers.

To reduce organizational barriers, parents suggested introducing case managers that might help parents and children with bridging the gaps between different healthcare professionals and in dealing with bureaucratic difficulties. Over the past year, several paediatric palliative care teams in the Netherlands have introduced such case managers, with the aim of trying to reduce this barrier. However, it is worth mentioning that in current practice, palliative care is predominantly limited to terminally ill children, and consequently, these palliative care services also remain limited to the terminal phase. However, issues with the continuity of care and with bureaucracy may arise long before this phase.

With regard to the third barrier, decision-making, the involvement of parents in decision-making varied. Previous studies also show that parents are not always involved in decision-making.[35-36] However, while these studies illustrate that some parents do not wish to be involved in decision-making,[37-38] all parents in our study expressed the importance of being involved in decision-making, with the only exception of decisions that involved merely medical knowledge. These differences may be explained by the different timing of decision-making processes (critical care versus longer term care). It is also possible that the

For peer review only - http://bmjopen.bmj.com/site/about/guidelines.xhtml

understanding of parents on what it means to be involved differs between studies (for example, being heard versus being fully responsible).

Shared decision-making has been an important movement in both paediatrics and medicine in general over the last decade.[39-40] Dutch regulations underline the importance of making parents, and – if possible – even children themselves, part of the decision-making process.[41] The fact that parents often feel excluded may be an incentive to further investigate why parents are not always included, and what is needed to achieve this.

The perceived need for options for active end of life decisions (e.g. euthanasia, or active life-ending on request of parents) is a barrier that, to our knowledge, has not previously been mentioned in studies on barriers in care for children with life-threatening conditions. In contrast, other studies mention parents' discomfort with making end-of-life decisions such as withholding or withdrawing treatment.[42] Although the Netherlands is known for having a legal regulation of euthanasia and end-of-life decision-making in adults and newborns,[25,43-44] the current regulations for children aged between one and 12 years old are similar to those in most other countries. Withholding and withdrawing treatment and palliative sedation are permitted, but active life-ending (euthanasia) is not. Following the Belgian legalization of euthanasia for competent minors, there has been a debate in the Netherlands about whether regulations on euthanasia should be broadened to allow active life-ending for children younger than 12 in cases of unbearable and hopeless suffering.[45-46]

Parents perceived a lack of care for the family. Family care is one of the goals of palliative care[8] and is arguably a service that all parents and siblings of the child should receive.[18] Previous studies highlight the importance of family support, including bereavement care.[12-13,17]

Previous studies have underlined that care for young children includes attention for a child's development.[18,47] Our study adds that this does not only mean seeing the child as a whole, with both physical, social and emotional aspects, but that it also means seeing the child as a *person*. From the perspective of parents, care for children with life-threatening conditions focuses too much on symptoms and not enough on the human beings behind the symptoms. This raises the question of whether such an attitude should be part of medical professionalism.

For peer review only - http://bmjopen.bmj.com/site/about/guidelines.xhtml

Medical professionalism: dehumanization?

Medicine and emotional intimacy seem to have a difficult relationship. Can a professional working in paediatric palliative medicine be expected to build an empathic, intimate relationship with all of their patients? Some authors argue that what makes a good doctor is, in essence, expertise and knowledge, not empathy; that good doctor may be stand-offish or surly, as long as they treat a symptom adequately.[48] Intimacy is a tool needed to detect the needs of a patient, but should be used very carefully, otherwise a doctor might 'lose himself".[48-50] Nevertheless, empathy benefits patients.[51] The view of detachedness as a professional quality is often – although not always explicitly – reiterated throughout medicine. There is a tension between this viewpoint of professional detachedness and that of empathic intimacy, or human-to-human contact, that parents ask for.

What parents seem to miss in current practise is the other connotation of the word 'care': to care about. We cannot expect physicians or nurses to become emotionally attached to every patient that they meet, but this is not what parents seem to miss in current care. What they miss in care is not the lack of showing emotion, but the affirmation that all parties – children, family and physicians – are in fact human and that the illness affects lives, not monitors. This attitude can be implemented into standard care for children with life-threatening conditions.

Medical detachedness versus attention for persons is not black or white, but rather a continuum, ranging from patient-physician relationships that focus on very specific symptoms, without much attention for the person who is experiencing it, towards recognizing the complexity of the child's overall health situation, and finally evaluating care in the context of a child who as a human being experiences its illness. In different forms of medicine different levels of detachedness, and attention for the person may be appropriate. A dental appointment, where the physician-patient relationship is short, and the treatment limited to a specific expertise, calls for a different kind of professionalism than paediatric palliative care, where its very goal implies attention for the person. By its goal –improving quality of life by relieving suffering–[2] paediatric palliative care has shifted its focus from the illness and symptom towards the patient. The experienced barriers indicate that this shift is possibly insufficiently translated into care. Perhaps it is time to consider how we should evaluate professional detachedness in the light of the goals of palliative care. Above all, policies and

For peer review only - http://bmjopen.bmj.com/site/about/guidelines.xhtml

guidelines should not merely guide professionals on the technical aspects of symptom treatment, but aid them in topics of professional intimacy as well.

Strengths and limitations.

Our study has some limitations. First, our study focused on young children (aged one to 12 years old) and the results cannot be generalized to adolescents or neonates. Second, although we put considerable effort into recruiting participants with different cultural and religious backgrounds, the success in achieving this variation was limited. Finally, we did not include the perspectives of physicians, in order to solely present the barriers perceived by parents. Although the parents' own experiences are the experiences that they live by, and that represent their reality, they do not present a complete overview alone. In order to fully evaluate care for children with life-threatening conditions, the reality of the physicians, nurses and other involved parties should also be described.

The strengths of this study are the size of the study. We held in-depth interviews with 64 participating parents, and over 110 hours of qualitative data on audio were collected. This makes the study unique in its size. A second strength is the variety. Most existing research is limited either by medical diagnosis or hospital where care is administered. In this study we interviewed parents from children with various illnesses, in various stages of their illness, receiving care from various hospitals, providing a much needed broad perspective on care given to children living with life-threatening conditions.

Conclusion

This study explores the barriers that parents encounter during the process of caring for children with life-threatening conditions. The barriers experienced by parents in care for children with life-threatening conditions and uncertain futures are related to six major themes. First, parents wished for more empathetic and open communication about the illness and prognosis. Second, organizational barriers create bureaucratic obstacles and a lack of continuity of care. Third, parents wished for more involvement in decision-making. Fourth, parents wished they had more support from the health care team on end-of-life decision-making. Fifth, parents experienced a lack of attention for the family during the illness and after the death of their child. Finally, parents experience an overemphasis on symptom-treatment and lack of attention for their child as a person. The parents' wish to see the child as a person sheds new light on the relationship between medical professionalism and detachedness. We argue that in paediatric palliative care, the child behind the symptoms is

For peer review only - http://bmjopen.bmj.com/site/about/guidelines.xhtml

sometimes forgotten. Paediatric palliative care might suit the needs of parents and children better when it re-evaluates its current professional detachedness, and progresses towards a medical professionalism where not only symptoms, but also people are treated.

Acknowledgements.

The authors would like to thank the participants for sharing their stories, the members of the advisory group and Veerle van de Wetering for their help in shaping the study and heir help in interpreting the results, and Suzanna Miedema-Wilsterman for her efforts in recruiting participants for this study.

Contributorship statement

All authors are responsible for the reported research, and article and meet the ICMJE standards for authorship. All authors agree to be accountable for all aspects of the work in ensuring that questions related to the accuracy or integrity of any part of the work are appropriately investigated and resolved.

MB contributed to the study design, conducted the recruitment and interviews, performed the qualitative analysis and drafted the manuscript.

EM contributed to the study design, helped in recruiting participants, performed the qualitative analysis, and contributed to the writing of the manuscript.

AvdH contributed to the study design, checked the qualitative analysis, and contributed to the writing of the manuscript.

IH contributed to the study design, contributed to the study design, checked the qualitative analysis, and contributed to the writing of the manuscript.

EV contributed to the study design, helped in recruiting participants, checked the qualitative analysis, and contributed to the writing of the manuscript.

Competing interest statement

The authors have no conflict of interest or financial relations to disclose.

Funding

This work was supported by 'Ministerie van Volksgezondheid, Welzijn en Sport'. Grant number 324717 (560019968)].

For peer review only

Data availability statement

There is no Additional Data Available.

Ethics approval

The study was reviewed and deemed exempt by the medical ethical committee of the University Medical Center Groningen. Number: METC2016. 223.

Figure legend/supplemental files

Figure 1. Participants' places of residence

Topic guide.

References

1. Mack JW, Wolfe J. Early integration of pediatric palliative care: for some children, palliative care starts at diagnosis. Curr Opin Pediatr 2006;18:10-4. doi:10.1097/01.mop.0000193266.86129.47 pmid:16470155
2. World Health Organization. WHO definition of palliative care. [cited 2019 Oct 08]. Available from: https://www.who.int/cancer/palliative/definition/en/
3. Brouwer M, Maeckelberghe E, De Weerd W, et al. Quality of Living and Dying: Pediatric Palliative Care and End-of-Life Decisions in the Netherlands. Camb Q Healthc Ethics 2018;27:376-84. doi:10.1017/S0963180117000767 pmid:29845907
4. Paediatric Association of the Netherlands (NVK). Richtlijn Palliatieve Zorg voor Kinderen. 2018 [cited 2019 Oct 08]. Available from: http://richtlijnendatabase.nl/richtlijn/palliatieve_zorg_voor_kinderen
5. International Children's Palliative Care Network (ICPCN) What is children's palliative care? 2010. [cited 2019 Oct 08]. Available from: http://www.icpcn.org.uk/page.asp?section=0001000100080004§ionTitle=What+is+Children%92s+Palliative+Care%3F
6. Sheetz MJ, Bowman MA. Pediatric palliative care: an assessment of physicians' confidence in skills, desire for training, and willingness to refer for end-of-life care. American Journal of Hospice and Palliative Medicine 2008;25:100-5. doi:10.1177/1049909107312592 pmid:18445861
7. Davies B, Sehring SA, Partridge JC, et al. Barriers to palliative care for children: perceptions of pediatric health care providers. J. Pediatr 2008;121:282-8. doi:10.1542/peds.2006-3153 pmid:18245419
8. Beckstrand RL, Rawle NL, Callister L, et al. Pediatric nurses' perceptions of obstacles and supportive behaviors in end-of-life care. Am J Crit Care. 2010;19:543-52 doi:10.4037/ajcc2009497 pmid:20026650

For peer review only - http://bmjopen.bmj.com/site/about/guidelines.xhtml

9. Gowan D. End-of-life issues of children. Pediatr Transplant 2003;7:40-3. doi:10.1034/j.1399-3046.7.s3.5.x pmid:12603691

10. Bartel DA, Engler AJ, Natale JE, et al. Working with families of suddenly and critically ill children: physician experiences. Arch Pediatr Adolesc Med 2000;154:1127-33. doi:10.1001/archpedi.154.11.1127 pmid:11074855

11. Williams‐Reade J, Lamson AL, Knight SM, et al. Paediatric palliative care: A review of needs, obstacles and the future. J Nurs Manag 2015;23:4-14. doi:10.1111/jonm.12095 pmid:23944156

12. Davies B, Connaughty S. Pediatric end-of-life care: Lessons learned from parents. J Nurs Adm 2002;32:5-6. doi:10.1097/00005110-200201000-00001 pmid:11802640

13. Contro N, Larson J, Scofield S, et al. Family perspectives on the quality of pediatric palliative care. Arch Pediatr Adolesc Med 2002;156:14-9. doi:10.1001/archpedi.156.1.14 pmid:11772185

14. Tenniglo LJ, Loeffen EA, Kremer LC, et al. Patients' and parents' views regarding supportive care in childhood cancer. Support Care Cancer 2017;25:3151-60. doi:10.1007/s00520-017-3723-7 pmid:28456909

15. Nyborn JA, Olcese M, Nickerson T, et al. "Don't try to cover the sky with your hands": parents' experiences with prognosis communication about their children with advanced cancer. J Palliat Med 2016;19:626-31. doi:10.1089/jpm.2015.0472 pmid:27115314

16. Hsiao JL, Evan EE, Zeltzer LK. Parent and child perspectives on physician communication in pediatric palliative care. Palliat Support Care 2007;5:355-65. doi:10.1017/S1478951507000557 pmid:18044413

17. Widger K, Picot C. Parents' perceptions of the quality of pediatric and perinatal end-of-life care. Pediatr Nurs 2008;34;53-8.

18. Goldman A. ABC of palliative care: special problems of children. BMJ. 1998;316:49–5. doi: 10.1136/bmj.316.7124.49 pmid:9451269

19. Hynson JL, Gillis J, Collins JJ, et al. The dying child: how is care different? Med J Aust. 2003;179:20–2. doi:10.5694/j.1326-5377.2003.tb05571.x pmid:12964930

20. Mack JW, Wolfe J. Early integration of pediatric palliative care: for some children, palliative care starts at diagnosis. Curr Opin Pediatr 2006;18:10-4. doi:10.1097/01.mop.0000193266.86129.47 pmid:16470155

21. Paediatric Association of the Netherlands (NVK). oproep melden casus levenseindezorg 1-12 jaren. 2016 [cited 2019 Oct 08]. Available from: https://www.nvk.nl/Nieuws/articleType/ArticleView/articleId/1707/Oproep-melden-casus-levenseindezorg-1-12-jarigen

22. Brouwer M. Maeckelberghe E, Verhagen E. Beslissingen en Zorg voor Kinderen met een Levenbedreigende Aandoening, Project Website. 2017, [cited 2019 Oct 08]. Available from: https://levensbedreigendziekkind.wordpress.com

23. Sandelowski M. What's in a name? Qualitative description revisited. *Res Nurs Health* 2010;33:77-84

24. Hennink M, Hutter I, Bailey A. Qualitative research methods. Thousand Oaks, CA, Sage; 2010 Dec 8

25. Government of the Netherlands. Wet toetsing levensbeëindiging op verzoek en hulp bij zelfdoding. [Termination of life on request and assisted suicide (Review Procedures) Act]. 2014. [cited 2019 Oct 08]. Available from: http:// wetten.overheid.nl/BWBR0012410/2014-02-15

26. Paediatric Association of the Netherlands (NVK). Richtlijn Palliatieve Zorg voor Kinderen. 2018. [cited 2019 Oct 08]. Available from: http://richtlijnendatabase.nl/richtlijn/palliatieve_zorg_voor_kinderen

27. Meyer EC, Sellers DE, Browning DM, et al. Difficult conversations: improving communication skills and relational abilities in health care. Pediatr Crit Care Med 2009;10:352-9. doi:10.1097/PCC.0b013e3181a3183a pmid:19325506

28. Hancock K, Clayton J, Parker S, et al. Truth telling in discussing prognosis in advanced life-limiting illnesses: a systematic review. Palliat Med. 2007;21:507-17. doi: 10.1177/0269216307080823 pmid:17846091

29. Crosnier-Schoedel C, Trocmé N, Carbajal R, et al. Pediatrician's experience in announcing bad news. Arch Pediatr. 2018;25:100-6. doi: 10.1016/j.arcped.2017.11.006 pmid:29254736

30. Baile WF, Lenzi R, Kudelka AP, et al. Improving physician—patient communication in cancer care: Outcome of a workshop for oncologists. J Cancer Educ 1997;12:166-73.

31. Coad J, Patel R, Murray S. Disclosing terminal diagnosis to children and their families: Palliative professionals' communication barriers. Death Stud 2014;38:302-7. doi: 10.1080/07481187.2012.753555 pmid:24593008

32. Afonso SB, Minayo MC. Relationships between oncohematopediatrics, mothers and children in communicating bad news. Cien Saude Colet 2017;22:53-62. doi: 10.1590/1413-81232017221.14592016 pmid:28076529

33. Kassam A, Skiadaresis J, Alexander S, et al. Differences in end-of-life communication for children with advanced cancer who were referred to a palliative care team. Pediatr Blood Cancer 2015;62:1409-13. doi: 10.1002/pbc.25530 pmid:25882665

34. Bally JM, Smith NR, Holtslander L, et al. A metasynthesis: Uncovering what is known about the experiences of families with children who have life-limiting and life-threatening illnesses. J Pediatr Nurs 2018;38:88-98. doi: 10.1016/j.pedn.2017.11.004 pmid:29357986

35. Barry MJ, Edgman-Levitan S. Shared decision making—the pinnacle of patient-centered care. N Engl J Med 2012;366:780-1. doi:10.1056/NEJMp1109283 pmid:22375967

36. Zaal-Schuller IH, Willems DL, Ewals FVPM, et al. How parents and physicians experience end-of-life decision-making for children with profound intellectual and multiple disabilities. Res Dev Disabil 2016;59:283-93. doi:10.1016/j.ridd.2016.09.012 pmid:27665411

37. de Vos MA, Bos AP, Plötz FB, et al Talking with parents about end-of-life decisions for their children. Pediatrics. 2015;135(2):e465-76. doi:10.1542/peds.2014-1903 pmid: 25560442

38. Madrigal VN, Carroll KW, Hexem KR, et al. Parental decision-making preferences in the pediatric intensive care unit. Crit care med. 2012;40(10):2876-82. doi:10.1097/CCM.0b013e31825b9151 pmid:22824932

For peer review only - http://bmjopen.bmj.com/site/about/guidelines.xhtml

39. Badarau DO, Ruhe K, Kühne T, et al. Decision making in pediatric oncology: Views of parents and physicians in two European countries. AJOB Empir Bioeth. 2017;8:21-31. doi:10.1080/23294515.2016.1234519 pmid:28949870

40. Kane HL, Halpern MT, Squiers LB, et al. Implementing and evaluating shared decision making in oncology practice. CA Cancer J Clin 2014;64:377–88. doi:10.3322/caac.21245 pmid:25200391

41. Paediatric Association of the Netherlands (NVK). Richtlijn Palliatieve Zorg voor Kinderen [Guidelines on Palliative Care for Children]. 2018 [cited 2019 Oct 08]. Available from: http://richtlijnendatabase.nl/richtlijn/palliatieve_zorg_voor_kinderen

42. Beckstrand RL, Rawle NL, Callister L, et al. Pediatric nurses' perceptions of obstacles and supportive behaviors in end-of-life care. Am J Crit Care 2010;19:543-52. doi:10.4037/ajcc2009497 pmid:20026650

43. Van der Heide A, Onwuteaka-Philipsen BD, Rurup ML, et al. End-of-life practices in the Netherlands under the Euthanasia Act. N Engl J Med 2007;356:1957-65. doi:10.1056/NEJMsa071143 pmid:17494928

44. Verhagen E, Sauer PJ. The Groningen protocol—euthanasia in severely ill newborns. N Engl J Med 2005;352:959-62. doi: 10.1056/NEJMp058026 pmid:15758003

45. Paediatric Association of the Netherlands (NVK). Standpunt NVK Levensbeëindiging kinderen 1-12 jaar: een pleidooi voor onderzoek. 2015. [cited 2019 Oct 08]. Available from: https://www.rijksoverheid.nl/documenten/publicaties/2016/04/22/standpunt-nvk-levensbeeindiging-kinderen-1-12-jaar-een-pleidooi-voor-onderzoek

46. Government of the Netherlands. Kamerbrief met reactie op NVK standpunt levensbeëindiging kinderen. 2016. [cited 2019 Oct 08]. Available from: https://www.rijksoverheid.nl/documenten/kamerstukken/2016/04/22/kamerbrief-met-reactie-op-nvk-standpunt-levensbeeindiging-kinderen

47. Rushton C. Integrating ethics and palliative care in pediatrics. Am J Nurs 2004;104:54–63. doi: 10.1097/00000446-200404000-00019 pmid:15171116

48. Hilhorst M, Deugden van de arts. In: Sanderse W, Kole J, eds. Karakter, Deugden voor professionals. Leusden: ISVW uitgevers 2018:103-12

49. Cheng Y, Lin CP, Liu HL, et al.Expertise modulates the perception of pain in others. Curr Biol. 2007;17:1708-13. doi:10.1016/j.cub.2007.09.020 pmid:17900903

50. Haque OS, Waytz A. Dehumanization in medicine: Causes, solutions, and functions. Perspect Psychol Sci. 2012;7:176-86. doi: 10.1177/1745691611429706 pmid:26168442

51. Haslam N. Humanising medical practice: the role of empathy. Med j Aust. 2007;187:381-2. doi: 10.5694/j.1326-5377.2007.tb01305.x pmid:17907998

For peer review only - http://bmjopen.bmj.com/site/about/guidelines.xhtml

Figure 1. Participants' places of residence

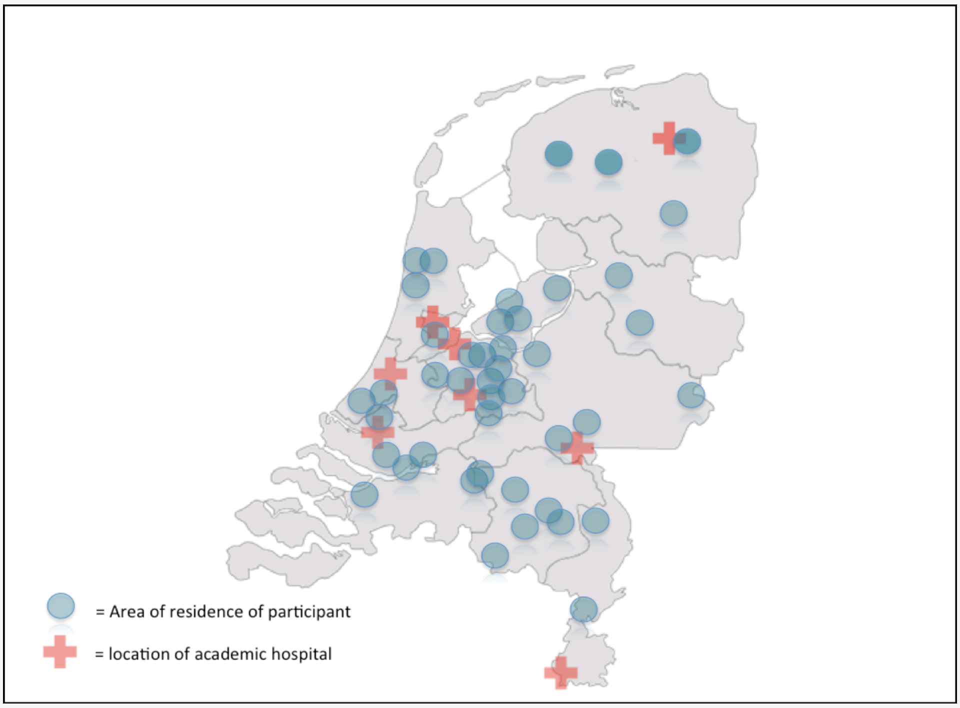

For peer review only

Appendix: Topic guide

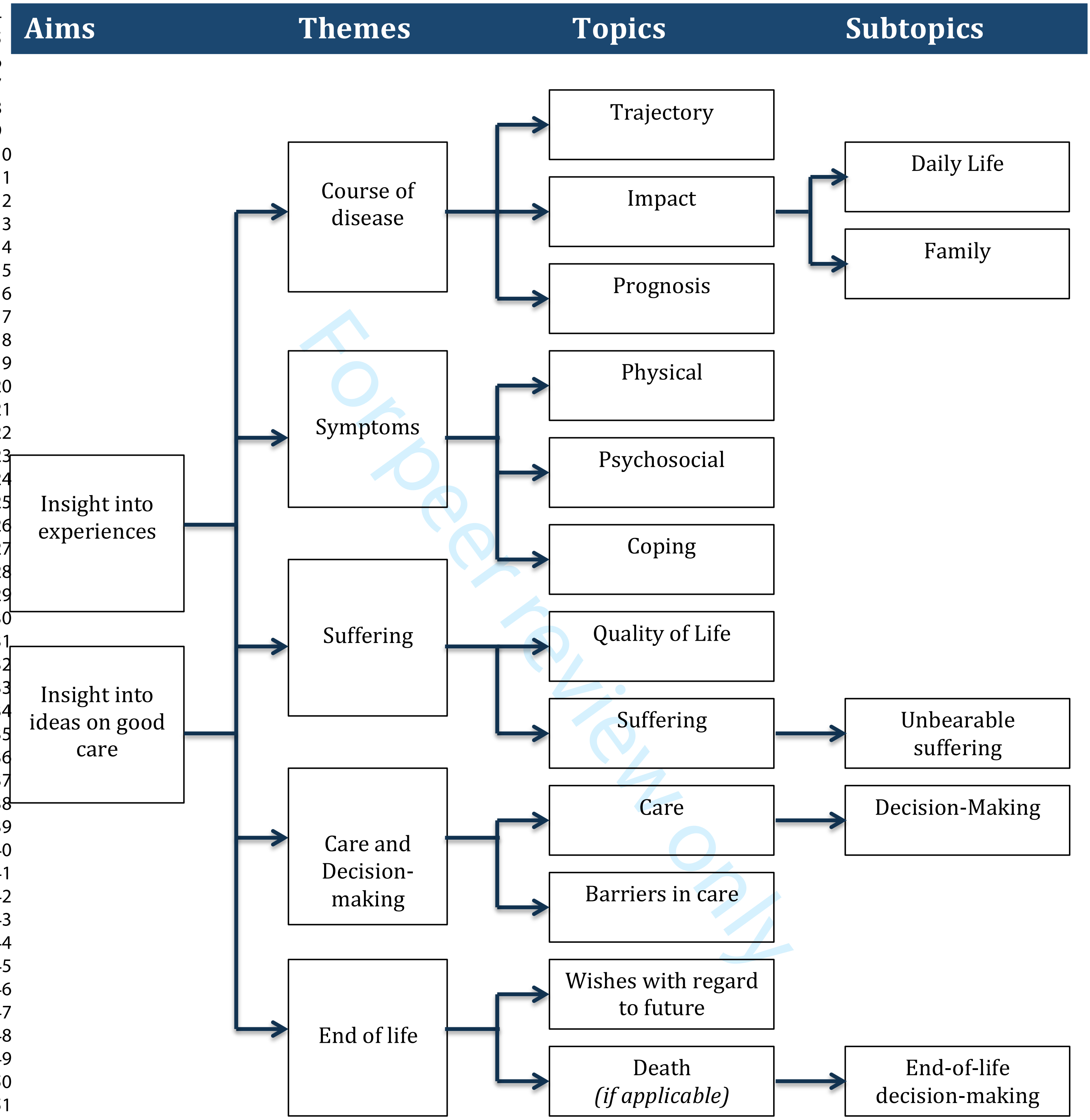

For peer review only

Consolidated criteria for reporting qualitative studies (COREQ): 32-item checklist

Developed from:
Tong A, Sainsbury P, Craig J. Consolidated criteria for reporting qualitative research (COREQ): a 32-item checklist for interviews and focus groups. *International Journal for Quality in Health Care*. 2007. Volume 19, Number 6: pp. 349 – 357

YOU MUST PROVIDE A RESPONSE FOR ALL ITEMS. ENTER N/A IF NOT APPLICABLE

No. Item	Guide questions/description	Reported on Page #
Domain 1: Research team and reflexivity		
Personal Characteristics		
1. Interviewer/facilitator	Which author/s conducted the inter view or focus group?	First author Methods, p. 5
2. Credentials	What were the researcher's credentials? E.g. PhD, MD	MA Methods, p.5
3. Occupation	What was their occupation at the time of the study?	PhD student Methods, p.5
4. Gender	Was the researcher male or female?	Female Methods, p.5
5. Experience and training	What experience or training did the researcher have?	training Methods, p.5
Relationship with participants		
6. Relationship established	Was a relationship established prior to study commencement?	none Methods, p.5
7. Participant knowledge of the interviewer	What did the participants know about the researcher? e.g. personal goals, reasons for doing the research	Nothing Methods, p.5
8. Interviewer characteristics	What characteristics were reported about the inter viewer/facilitator? e.g. Bias, assumptions, reasons and interests in the research topic	None Methods, p.5
Domain 2: study design		
Theoretical framework		
9. Methodological orientation and Theory	What methodological orientation was stated to underpin the study? e.g. grounded theory, discourse analysis, ethnography, phenomenology, content analysis	Thematic analysis using constant comparative method. Methods, p.6
Participant selection		
10. Sampling	How were participants selected? e.g. purposive, convenience, consecutive, snowball	Maximum variety Methods, p.5
11. Method of approach	How were participants approached? e.g. face-to-face, telephone, mail, email	Various. See Methods, p.5

For peer review only

12. Sample size	How many participants were in the study?	64 parents of 44 children.<br><br>Results, see p.6
13. Non-participation	How many people refused to participate or dropped out? Reasons?	3, Methods. p.5
Setting		
14. Setting of data collection	Where was the data collected? e.g. home, clinic, workplace	Methods, interviews. p. 5
15. Presence of non-participants	Was anyone else present besides the participants and researchers?	Some parents were interviewed together with their spouse.<br>p.5
16. Description of sample	What are the important characteristics of the sample? e.g. demographic data, date	See Results, table 1 and 2. (p.6, p.7)
Data collection		
17. Interview guide	Were questions, prompts, guides provided by the authors? Was it pilot tested?	Methods, interviews see p.2
18. Repeat interviews	Were repeat inter views carried out? If yes, how many?	No.<br>Methods, p.5
19. Audio/visual recording	Did the research use audio or visual recording to collect the data?	Audio records<br><br>Methods, p.5
20. Field notes	Were field notes made during and/or after the inter view or focus group?	N/A
21. Duration	What was the duration of the inter views or focus group?	On average 2 hours.<br><br>Methods, p.5
22. Data saturation	Was data saturation discussed?	Yes<br>Methods, p.5
23. Transcripts returned	Were transcripts returned to participants for comment and/or correction?	Yes,<br>Methods. p.5
Domain 3: analysis and findings		
Data analysis		
24. Number of data coders	How many data coders coded the data?	Two main coders, checked by all 5 authors.<br>Methods see p.5
25. Description of the coding tree	Did authors provide a description of the coding tree?	See results, p.6. The coding tree can be added to the manuscript if needed.
26. Derivation of themes	Were themes identified in advance or derived from the data?	From the data.<br>See methods. p.5
27. Software	What software, if applicable, was used to	Atlas.ti,

For peer review only

	manage the data?	Methods, data analysis, see p.5
28. Participant checking	Did participants provide feedback on the findings?	By means of an advisory board.<br><br>Methods, p.6
Reporting		
29. Quotations presented	Were participant quotations presented to illustrate the themes/findings? Was each quotation identified? e.g. participant number	Yes, See results.
30. Data and findings consistent	Was there consistency between the data presented and the findings?	See results and discussion
31. Clarity of major themes	Were major themes clearly presented in the findings?	See Results
32. Clarity of minor themes	Is there a description of diverse cases or discussion of minor themes?	See results.

For peer review only

Standards for Reporting Qualitative Research (SRQR)

O'Brien B.C., Harris, I.B., Beckman, T.J., Reed, D.A., & Cook, D.A. (2014). Standards for reporting qualitative research: a synthesis of recommendations. *Academic Medicine, 89(9)*, 1245-1251.

No. Topic	Item
**Title and abstract**	
S1 Title<br><br>Concise description of the nature and topic of the study identifying the study as qualitative or indicating the approach (e.g., ethnography, grounded theory) or data collection methods (e.g., interview, focus group) is recommended	The Title of the manuscript is:<br><br>*Barriers in Care for Children With Life-Threatening Conditions: a Qualitative Interview Study in he Netherlands*
S2 Abstract<br><br>Summary of key elements of the study using the abstract format of the intended publication; typically includes objective, methods, results, and conclusions	The key elements of the study (objective, design, setting, participants results and conclusions) have been summarized in the abstract. (see p.2)
**Introduction**	
S3 Problem formulation<br><br>Description and significance of the problem/phenomenon studied; review of relevant theory and empirical work; problem statement	The main problem is that in current literature on pediatric palliative care, knowledge of parents' experiences of barriers to good pediatric palliative care is limited. This is stated in the introduction (see p.2)
S4 Purpose or research question<br><br>Purpose of the study and specific objectives or questions	The purpose of the study is to provide insight into barriers to care and decision-making for children living with life-threatening conditions. (see p.2)
**Methods**	
S5 Qualitative approach and research paradigm<br><br>Qualitative approach (e.g., ethnography, grounded theory, case study, phenomenology, narrative research) and guiding theory if	The qualitative approach used in this study is qualitative description, and a thematic analysis was performed using the constant comparative approach. (see p.6)

For peer review only

appropriate; identifying the research paradigm (e.g., positivist, constructivist/interpretivist) is also recommended	
S6 Researcher characteristics and reflexivity<br><br>Researchers' characteristics that may influence the research, including personal attributes, qualifications/experience, relationship with participants, assumptions, or presuppositions; potential or actual interaction between researchers' characteristics and the research questions, approach, methods, results, or transferability	Characteristics of the interviewer are described in methods section interviews (see p.5)<br>Credentials:MA<br>Position: PhD student<br>Sex: female<br>education in interviewing: formal training<br><br>The analysis team consisted of a PhD student, an ethicist, a professor in end-of-life care, and a child psychiatrist and a physician/professor in paediatric palliative care. This is described in the methods section (see p.6)
S7 Context<br><br>Setting/site and salient contextual factors; rationale[a]	Participants were usually interviewed at their place of residence. They were free in conducting the interview alone or together (see p.5)
S8 Sampling strategy<br><br>How and why research participants, documents, or events were selected; criteria for deciding when no further sampling was necessary (e.g., sampling saturation); rationale[a]	. Sampling was aimed at obtaining maximum variety in terms of health condition, age, cultural background, parental level of education and place of residence. (see p.5)<br><br>To recruit participants, all Dutch paediatricians received an invitation from the Paediatric Association of the Netherlands (NVK) to invite potential parents to join the research. A website with information about the study was created to inform potential participants. Parent support groups in oncology, cardiology, metabolic and neuromuscular diseases and palliative care helped to reach potential participants through their online platforms. To ensure maximum variety in ethnic background we recruited through physicians with expertise in treating patients with different cultural backgrounds. (see p.5)<br><br>Sampling continued until thematic saturation and saturation of variety in participants was achieved. (see p.5)
S9 Ethical issues pertaining to human subjects<br><br>Documentation of approval by an appropriate ethics review board and participant consent, or explanation for lack thereof; other confidentiality and data security issues	The study was reviewed and deemed exempt by the medical ethical committee of the University Medical Center Groningen. (see p.5)
S10 Data collection methods<br><br>Types of data collected; details of data collection procedures including (as appropriate) start and stop dates of	Parents were interviewed in face-to-face in-depth interviews.

For peer review only

data collection and analysis, iterative process, triangulation of sources/methods, and modification of procedures in response to evolving study findings; rationale[a]	
S11 Data collection instruments and technologies<br><br>Description of instruments (e.g., interview guides, questionnaires) and devices (e.g., audio recorders) used for data collection; if/how the instrument(s) changed over the course of the study	A topic guide had been developed as an interview guide. The interview covered the following themes: course of the disease, aspects of the illness (symptoms and impact) suffering, care, decision-making and end of life. The topic guide is added as a supplementary file to the manuscript. (see p.5)
S12 Units of study<br><br>Number and relevant characteristics of participants, documents, or events included in the study; level of participation (could be reported in results)	In the results section, the relevant characteristics of the participants are described: characteristics of children: status, age, gender, diagnosis<br>Characteristics of parents: relationship status, education, ethnicity, spiritual beliefs, family composition.<br><br>(see p.7 table 1 and 2)
S13 Data processing<br><br>Methods for processing data prior to and during analysis, including transcription, data entry, data management and security, verification of data integrity, data coding, and anonymization/deidentification of excerpts	The interviews were recorded on audio and subsequently transcribed verbatim. All interviews were anonymized during transcription. Participants were given the opportunity to revise their transcribed interviews. (see. p.5)
S14 Data analysis<br><br>Process by which inferences, themes, etc., were identified and developed, including researchers involved in data analysis; usually references a specific paradigm or approach; rationale[a]	A primary thematic analysis, aiming at a qualitative description of barriers as perceived by parents, was performed using the constant comparative approach.
S15 Techniques to enhance trustworthiness<br><br>Techniques to enhance trustworthiness and credibility of data analysis (e.g., member checking, audit trail, triangulation); rationale[a]	Coding was performed by the first two authors and reviewed by all authors. Differences were settled by discussion until consensus was achieved. During the study, biannual meetings were held with an advisory board (9 members) of parents, physicians and researchers who provided feedback on the findings. (see. p.6)
**Results/Findings**	
S16 Synthesis and interpretation<br><br>Main findings (e.g., interpretations, inferences, and themes); might include development of a theory or model, or integration with prior research or theory	As the theoretical framework of this study is qualitative decription, we did not include a theoretical model in this transcript. The results section discusses the main barriers as experienced by parents (see results section)
S17 Links to empirical data	In the results section, quotes are used to illustrate the findings. (see

For peer review only

Evidence (e.g., quotes, field notes, text excerpts, photographs) to substantiate analytic findings	results section)
**Discussion**	
S18 Integration with prior work, implications, transferability, and contribution(s) to the field<br><br>Short summary of main findings; explanation of how findings and conclusions connect to, support, elaborate on, or challenge conclusions of earlier scholarship; discussion of scope of application/generalizability; identification of unique contribution(s) to scholarship in a discipline or field	In the discussion, the main findings are discussed (see p.14) and placed in the context of exiting literature. (see p. 14- 17)
S19 Limitations<br><br>Trustworthiness and limitations of findings	The discussion contains a limitations section discussing relevant limitations of this study. (see p. 17)
**Other**	
S20 Conflicts of interest<br><br>Potential sources of influence or perceived influence on study conduct and conclusions; how these were managed	Disclosure of interests. The authors have no conflict of interest or financial relations to disclose. (see p. 18)
S21 Funding<br>Sources of funding and other support; role of funders in data collection, interpretation, and reporting	This work was supported by the Dutch Ministry of Health, Welfare and Sport. Grant number 324717 (560019968)] (see p.18)

[a]The rationale should briefly discuss the justification for choosing that theory, approach, method, or technique rather than other options available, the assumptions and limitations implicit in those choices, and how those choices influence study conclusions and transferability. As appropriate, the rationale for several items might be discussed together.

For peer review only
